# Plant-Derived Agents and Systemic Sclerosis: A Systematic Review of Therapeutic Potential and Molecular Mechanisms

**DOI:** 10.3390/cimb48010097

**Published:** 2026-01-18

**Authors:** Cristian-Mihai Ilie, Teodora-Cristiana Grădinaru, Cătălina Anamaria Boromiz, Marilena Gilca

**Affiliations:** 1 Department of Functional Sciences I/Biochemistry, Carol Davila University of Medicine and Pharmacy, 050474 Bucharest, Romania; cristian-mihai.ilie@rez.umfcd.ro (C.-M.I.); teodora-cristiana.gradinaru@umfcd.ro (T.-C.G.); 2 Department of Rheumatology and Internal Medicine, Sfânta Maria Clinical Hospital Bucharest, 011172 Bucharest, Romania; catalina.boromiz@drd.umfcd.ro

**Keywords:** systemic sclerosis, phytocompounds, plant, anti-inflammatory, antioxidant, antifibrotic, SwissTargetPrediction

## Abstract

Systemic sclerosis (SSc) is a rare multisystemic autoimmune disease associated with progressive fibrosis, vasculopathy, and immune dysregulation. Despite advances in its management, the disease remains associated with substantial morbidity and mortality, with limited therapeutic options. This systematic review aimed to identify phytocompounds and medicinal plants that had demonstrated efficacy in SSc. A comprehensive literature search was performed in PubMed and ScienceDirect, yielding 7797 records, of which 32 studies met the inclusion criteria. A second search was performed using the SwissTargetPrediction tool to identify new putative molecular targets for these phytocompounds, whose relevance for SSc pathogenesis was verified by a third search in PubMed and ScienceDirect databases. Our search found 24 phytocompouds (e.g., halofunginone, crocetin, and tanshinone IIA) and 5 plant extracts (e.g., caper bush and ciplukan) reported to modulate key pathogenic processes in SSc. These phytochemicals were mainly associated with effects on endothelial to mesenchymal transition, oxidative stress, inflammation, and profibrotic signaling pathways, particularly TGF-β/Smad. The SwissTargetPrediction tool indicated 93 new potential molecular targets of the selected phytochemicals, among which only 41 showed relevance to SSc pathogenesis. In conclusion, available evidence is scarce but promising. Further studies, especially human investigations, are required to clarify clinical efficacy, safety, and potential interactions with drugs used in SSc.

## 1. Introduction

Systemic sclerosis (SSc) is a complex autoimmune connective tissue disorder, which is characterized by progressive fibrosis of skin and internal organs and microvascular damage [1,2].

The global incidence of SSc is estimated at 1.4–8.6 cases per 100,000 person-years, with a prevalence range from 17.6 to 18.9 per 100,000 individuals. Although mortality at younger ages has declined, the overall mortality still remains elevated compared to the general population. The leading causes of death in SSc patients are interstitial lung disease (ILD) and pulmonary arterial hypertension (PAH) [3].

SSc is a clinically heterogeneous disease characterized by a wide spectrum of manifestations, including cutaneous (progressive skin thickening with sclerodactyly, microstomia, and joint contractures leading to deformities), vascular (Raynaud phenomenon, cutaneous telangiectasia, and ischemic digital ulcers), and visceral (e.g., cardiac, respiratory, renal, and gastrointestinal) manifestations [4].

The pathogenesis of SSc is currently understood as a triad consisting of endothelial injury, immune system activation with the production of specific autoantibodies, and progressive fibrosis, although its exact mechanisms still remain unclear [5].

According to the updated 2023 European Alliance of Associations for Rheumatology (EULAR) treatment recommendations, the management of SSc is individualized and tailored to organ-specific involvement. In Raynaud phenomenon, first-line therapy consists of dihydropyridine-type calcium channel antagonists (most commonly Nifedipine), with escalation to phosphodiesterase-5 (PDE5) inhibitors or intravenous Iloprost in refractory cases. Digital ulcers are treated with PDE5 inhibitors, intravenous Iloprost, or with the endothelin receptor antagonist—Bosentan. PAH requires early initiation of combination therapy with PDE5 inhibitors and endothelin receptor antagonists, additional prostacyclin analogues (e.g., Selexipag) or Riociguat where indicated, with consideration of Epoprostenol for patients with severe PAH. Scleroderma renal crisis mandates the prompt initiation of angiotensin-converting enzyme inhibitors. Gastrointestinal manifestations are addressed with proton pump inhibitors for reflux, prokinetic agents for dysmotility, and cyclical antibiotics in the setting of small intestinal bacterial overgrowth. Cutaneous fibrosis may be addressed with immunosuppressive therapies such as Methotrexate, Mycophenolate mofetil, Rituximab, or Tocilizumab. Management of ILD involves immunosuppressive therapies such as Mycophenolate mofetil, Cyclophosphamide, Rituximab, or Tocilizumab, and antifibrotic treatment with Nintedanib. Musculoskeletal manifestations are generally addressed through treatment with Methotrexate [6].

Despite substantial progress in the management of SSc over the past few decades, the disease continues to be associated with considerable morbidity and mortality. Current management protocols have greatly improved survival in SSc-related ILD and PAH, but they are still limited regarding Raynaud phenomenon, digital ulcers, cardiac, gastrointestinal, or renal involvement. In addition, a subset of patients shows limited responsiveness to existing therapeutic options and continues to demonstrate disease progression despite the appropriate therapy [7].

A survey on herbal therapies conducted with patients with SSc reported that 9 of the 23 patients used a variety of phytotherapy products (essential oils, herbal tea, vegetable oils, or gemmotherapy). On a rating scale of 1–10, the perceived effectiveness of these treatments was an average of 7.2 [8]. In light of these findings, this research sought to evaluate the available data on the therapeutic potential of plant-derived agents in SSc and to summarize the putative molecular mechanisms underlying their biological activity.

## 2. Materials and Methods

The first step of our analysis consisted of a comprehensive search of the scientific literature, which was conducted in PubMed and ScienceDirect, encompassing publications from January 1975 through August 2025. Each study we found was carefully reviewed and assessed for eligibility following the PRISMA 2020 guidelines to ensure a transparent and systematic selection process [9]. Our review was focused on molecular mechanisms of plant-derived agents, and the results were synthesized using a narrative approach supported by tabular summaries; consequently, given these aspects and the absence of quantitative data synthesis or meta-analysis, the review protocol was not registered in the International Prospective Register of Systematic Reviews (PROSPERO).

The inclusion criteria for this review were established though a systemic search strategy combining various keywords including (“systemic sclerosis” OR “systemic scleroderma” OR “scleroderma” OR “sclerosis”) AND (“plants” OR “herb” OR “phytocompound” OR “phytochemicals”) NOT (“multiple sclerosis” OR “fungus” OR “lateral sclerosis” OR “tuberous sclerosis”). Additionally, a linguistic eligibility criterion was implemented to include only studies published in the English language. Search strings applied across PubMed and ScienceDirect are provided in the Supplementary Materials.

The exclusion criteria encompassed duplicate publication, studies addressing a natural compound but not related to SSc, research on SSc unrelated to natural compound and articles referring to herbal remedies from traditional medicine that had not been scientifically evaluated for SSc.

The main characteristics of the studies included in this systematic review are presented in Supplementary Table S1. An abbreviated version of the Standard Quality Assessment Criteria by Kmet et al. [10] was used to evaluate the methodological quality of all studies. Risk of bias was assessed according to study design, using the Systematic Review Centre for Laboratory animal Experimentation (SYRCLE) Risk of Bias tool [11] for in vivo studies and the Risk Of Bias In Non-randomized Studies-of Interventions (ROBINS-I) tool for human studies [12]. The assessments were performed by one reviewer and independently checked by a second reviewer to minimize bias. The results are summarized in Supplementary Tables S2–S4.

Following the systematic literature search, we extracted all evidence-based molecular targets involved in SSc pathogenesis and generated a synthesized table summarizing the reported interactions and activities of the identified phytocompounds on these targets.

Next, we performed a second search by using the SwissTargetPrediction web tool (http://www.swisstargetprediction.ch/, accessed on 15 October 2025) to identify new molecular targets of the bioactive phytocompounds that were found in our first search.

SwissTargetPrediction is a bioinformatic tool developed to predict ligand–target affinity for small molecules [13]; therefore, the phytochemicals with molecules that are too large, with more than 200 characters per SMILES (e.g., asiaticoside and madecassoside), or enzymes (e.g., bromelain) could not be submitted to the SwissTargetPrediction tool. Furthermore, no target predictions were produced by SwissTargetPrediction for the phytochemical extracts (e.g., proanthocyanidins and astragalus polysaccharides). The target predictions were sorted from high to low according to probability score. Subsequent analyses were limited to those targets with a SwissTargetPrediction probability score above 0.5. The greater the probability score, the more accurate the predicted target; therefore, a probability above 0.5 is commonly set as the threshold to filter more credible targets in network pharmacology studies [14,15,16,17].

We also excluded from this list any molecular targets for which the designation “by homology” appeared after the target name, in order to mi minimize potential false-positive predictions.

We listed all the putative molecular targets of the phytochemicals. Afterwards we have compared this list with the list of evidence-based targets from the first search.

Finally, a third search was conducted in PubMed and ScienceDirect to determine whether these newly identified putative molecular targets of the bioactive phytochemicals may play a demonstrable role in the pathogenesis of SSc, by using the phrase: [(name of the molecular target) AND (“systemic sclerosis” OR “systemic scleroderma”, “scleroderma” OR “sclerosis”) NOT “multiple sclerosis” NOT “fungus” NOT “lateral sclerosis” NOT “tuberous sclerosis”].

**Figure 1:**
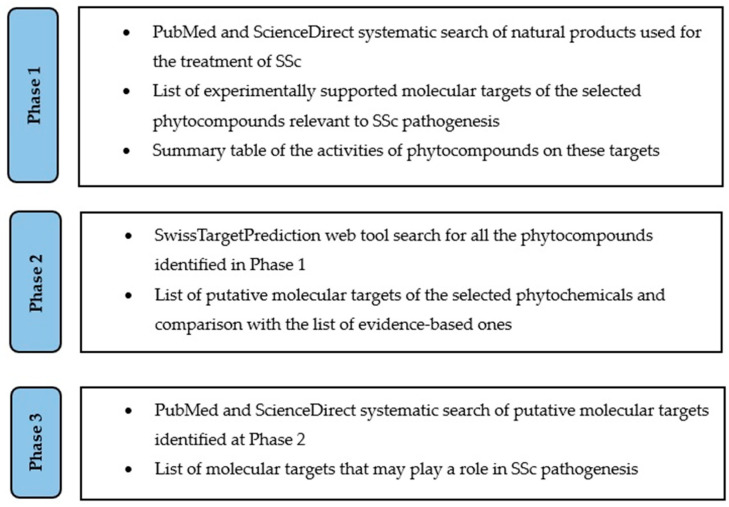
Phases of the study—summary.

The workflow of the study, organized into three phases, is summarized in Figure 1.

## 3. Results

**Figure 2:**
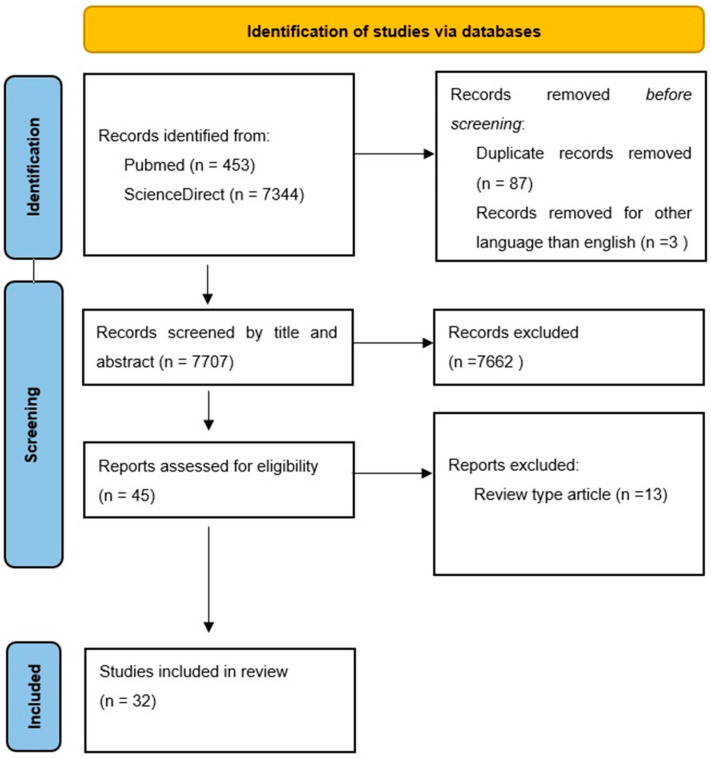
Preferred Reporting Items for Systematic Reviews and Meta-Analyses (PRISMA) flowchart.

The literature search identified 453 studies on PubMed and 7344 studies on ScienceDirect. Titles and abstracts were screened to remove duplicates and irrelevant records. Duplicates, irrelevancy, or unreliability resulted in exclusion of 7765 articles. Lastly, 32 studies (original or research articles) were selected for the review and were included in the synthesized tables (Table 1, Table 2, Table 3, Table 4, Table 5 and Table 6). The study selection process is illustrated using a PRISMA 2020 flow diagram (Figure 2), and a complete PRISMA checklist is provided [9].

A structured synthesis of the principal phytocompounds evaluated, their molecular targets, and the corresponding levels of evidence, stratified by study design, is provided in Table 7, allowing a clear and systematic overview of the available data.

To integrate the findings of our literature analysis, we compiled a list of evidence-based molecular targets of the selected phytocompounds relevant to SSc pathogenesis and developed a summary table detailing the documented action of the corresponding bioactive phytochemicals, resulting in a comprehensive color-coded phytocompound-target map covering 68 molecular targets involved in SSc pathogenesis (Supplementary Table S5). In this map, green-marked entries correspond to evidence-based molecular targets (e.g., direct inhibition or binding), orange entries indicate an evidence-based effect on genetic expression (e.g., modulation of mRNA levels), and blue entries denote an evidence-based modification of molecular levels (e.g., plasma or tissular levels).

To further extend the analytical scope of these findings, all phytocompounds were subsequently analyzed using the SwissTargetPrediction web tool, resulting in the identification of an additional set of 93 predicted molecular targets (Supplementary Table S6). In Supplementary Table S6, only the first two columns generated by SwissTargetPrediction were retained: the molecular target name and the common name (linked to GeneCards) [13]. Notably, only one target from the initial evidence-based list derived from the primary literature search (MAPK p38α) was also predicted by the SwissTargetPrediction.

The predicted molecular targets identified using the Swiss Target Prediction analysis (which were not listed in the first phase, except for MAPK p38 alpha) were subjected to a third literature search. This search confirmed the relevance of 43 out of the 93 predicted targets to SSc pathogenesis (Table 8).

## 4. Discussion

**Figure 3:**
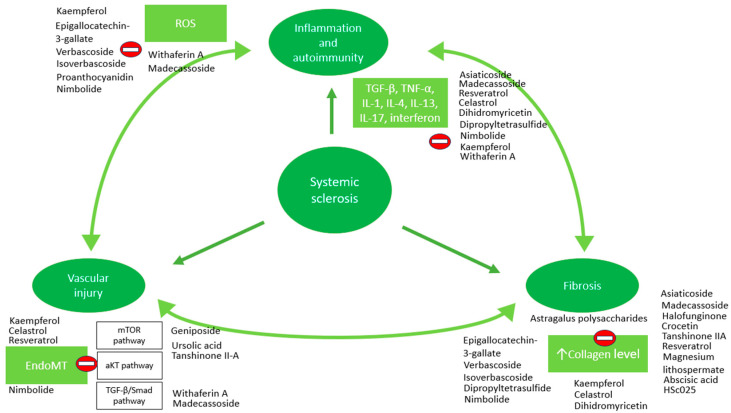
The pathogenic triad in SSc and phytocompounds therapeutic actions: aKT—protein kinase B; mTOR—mammalian target of rapamycin; TGF-β—transforming growth factor β; TNF-α—tumor necrosis factor α; ROS—reactive oxygen species.

In the context of autoimmunity, anti-centromere, anti-Th/To, and anti-topoisomerase I antibodies are recognized as classical biomarkers of SSc, reflecting disease-associated immune reactivity directed against nuclear components [105]. Upon exposure to pathological stimuli (TGF-β, TNF-α, IL-1, interferon, endothelin-1—ET-1, reactive oxygen species—ROS, hypoxia), endothelial cells undergo structural and functional changes, a process known as endothelial-to-mesenchymal transition (EndoMT) [106]. Through this process, endothelial cells adopt a myofibroblast phenotype, acquiring invasive properties, upregulating mesenchymal markers (α-smooth muscle active—α-SMA, CD44, N-cadherin, vimentin, smooth muscle 22—Sm22, and fibroblast-specific protein-1—FSP-1) and producing collagen [106]. Moreover, myofibroblasts could exacerbate the inflammation by upregulating adhesion molecules, thereby facilitating interactions with circulating immune cells, and by secreting growth factors and cytokines [107]. Fibrosis results from the excessive deposition of collagen and other extracellular matrix proteins by myofibroblasts, occurring in the skin, lungs, gastrointestinal tract, and other vital organs [108]. Subendothelial deposition of fibrous tissue promotes abnormal vascular remodeling, which increases capillary fragility and thereby sustains immune response activation [109]. Therefore, it can be stated that the three components of the pathogenic triad in SSc are in a constant and dynamic interrelationship (Figure 3).

There are several pathways involved in EndoMT, such as Smad-dependent and independent TGF-β pathways [110], mammalian target of rapamycin pathway (mTOR) [111], and protein kinase B pathway (aKT) [112].

During the EndoMT process, the cell loses endothelial cell biomarkers (VE-cadherin and CD31) [113] and upregulates the expression of mesenchymal cell biomarkers (α-SMA, N-cadherin, vimentin, FSP-1) [106]. Thus, as summarized in the results tables, the following phytocompounds have been reported to exert inhibitory effects on the EndoMT process: tanshinone II-A [21], resveratrol [22], geniposide [25], ursolic acid [37], nimbolide [41], withaferin A [42], kaempferol [31], celastrol [32], madecassoside [44], and 5-(tert-Butyl)-N-(1-hydroxy-2-methylpropan-2-yl)-1-(5-(trifluoromethyl)pyridin-2-yl)-1H-pyrazole-3-carboxamide [46]. Additionally, the ethanol extract of *Capparis spinosa* L., which contains flavonoids such as kaempferol, also induces inhibition of the EndoMT process [49]. Since no human studies have been reported for any of the aforementioned phytocompounds, further investigation of this mechanism in patients with SSc appears warranted.

Angiotensin II has been identified as a key inducer of EndoMT, contributing to fibrotic process progression [114]. Previous studies demonstrated that angiotensin II activate TGF-β signaling pathway, thereby promoting EndoMT and amplifying the inflammatory response through enhanced myofibroblast accumulation. This pathological cascade leads to excessive collagen synthesis and deposition in the skin [115]. Furthermore, co-expression of endothelial and mesenchymal markers in patients with interstitial lung disease (ILD) was observed, suggesting that EndoMT was involved in pathogenesis of ILD in SSc patients [116]. All these findings suggest that EndoMT has a role in the progression of fibrosis in SSc.

Across most studies, antifibrotic efficacy was assessed through the quantification of collagen. According to the data summarized in the tables, various phytocompounds have demonstrated antifibrotic activity in SSc models, including halofunginone [18], crocetin [19], magnesium lithospermate [23], dipropyltetrasulfide [24], tanshinone IIA [20,21], resveratrol [22], absicisic acid [28], HSc025 [30], epigallocatechin-3-gallate [33], curcumin [36], verbascoside [38], isoverbascoside [38], nimbolide [41], kaempferol [31], dihydromyricetin [34], astragalus polysaccharide [43], madecassoside [44], asiaticoside [45], and 5-(tert-Butyl)-N-(1-hydroxy-2-methylpropan-2-yl)-1-(5-(trifluoromethyl)pyridin-2-yl)-1H-pyrazole-3-carboxamide [46]. In addition, *Capparis spinosa* L., which contains flavonoids, such as Kaempferol, induces a similar effect [49].

pSmad2/3 is a key protein involved in TGFβ induced fibrosis [117]. Our search revealed that curcumin [36], verbascoside [38], isoverbascoside [38], dipropyltetrasulfide [24], nimbolide [41], withaferin A [42], and madecassoside [44] inhibit the phosphorylation, and therefore the activation of Smad2/3.

In the context of fibrosis, additional plant-derived agents have shown promising beneficial effects. Tanshinone II-A has already been traditionally used for treating patients with pulmonary [118] and liver fibrosis [119,120]. Results show that *Tripterygium wilfordii Hook.f.* confers pulmonary benefits evidenced by increases in mean FVC and FVC % of predicted, when it is used as a maintenance therapy. Considering that both in vivo and in vitro studies have demonstrated beneficial effects on pulmonary fibrosis for HSc025 [30], verbascoside [38], isoverbascoside [38], madecassoside [44], and asiaticoside [45], further investigation in patients with SSc and pulmonary involvement represents a novel and promising research direction.

Multiple phytocompounds (asiaticoside [45], resveratrol [22], celastrol [32], dihydromyricetin [34], dipropyltetrasulfide [24], nimbolide [41], withaferin A [42], kaempferol [31], madecassoside [44]) exerted anti-inflammatory effects in SSc models. One of the key players in the inflammation and fibrosis branches of the SSc pathogenesis triad, TNF-α, contributes to fibroblast activation and its expression has also been linked to the progression and severity of scleroderma [121].

In addition to the previously mentioned isolated phytocompounds, a few plant-derived whole or fractionated extracts were also tested, some proving various degrees of clinical efficacy even in human studies (e.g., caper bush extract [48,49], Lei Gong Teng [51]), while others not (e.g., St. John’s Wort [53]). Regarding St. John’s Wort, although this plant has not been demonstrated to improve the parameters of Raynaud’s phenomenon attacks [53], its content in flavonoids (kaempferol, luteolin) and tannins (proanthocyanidins) [52], which are already found to be bioactive in SSc, and its antioxidant potential [122], warrant further investigation in patients with SSc.

Evening primrose *(Oenothera biennis* L., family Onagraceae) seed oil is a rich source of gamma-linoleic acid [123]. Supplementation with *Oenothera biennis* L. oil in patients with SSc may alleviate pain in the hands and feet, promote digital ulcer healing, and improve telangiectasias and skin texture. The therapeutic effects are presumed to result from gamma-linoleic acid, which functions as a metabolic precursor of prostaglandin E1 [124]. Evidence regarding its efficacy in Raynaud phenomenon remains inconclusive [54,55].

Extract of *Ginkgo biloba* L. (family Ginkgoaceae), rich in flavonoids [125], has been demonstrated to attenuate endothelial-monocyte adhesion by reducing TNF-α-induced intracellular formation of reactive oxygen species (ROS) in human aortic endothelial cells [126]. Taking into account that the potent antioxidant capacity of flavonoids contributes to their therapeutic effect in oxidative stress-related dermatoses [127], it would be a reasonable assumption that this mechanism may also be involved in case of *Ginkgo biloba* L. use as an anti-SSC remedy. Elevated ROS contributes to tissue fibrosis by upregulating collagen type I and tissue inhibitor of metalloproteinase gene expressions and reducing the degradation of ECM [128]. Under these circumstances, future research investigating the effects of *Ginkgo biloba* L. on fibrotic processes in SSc appears warranted.

*Centella asiatica* (L.) Urb., a member of the Apiaceae family, contains a high amount of triterpene glycosides, primarily asiaticoside and madecassoside, which are the main bioactive compounds. Consequently, *Centella asiatica* extracts display notable antioxidant and anti-inflammatory properties, largely attributable to asiaticoside, asiatic acid, and madecassoside. These compounds mitigate oxidative stress by reducing malondialdehyde levels and enhancing glutathione levels [129].

Recent studies have demonstrated that the endocannabinoid system can modulate dysregulated mechanisms implicated in SSc pathogenesis, including fibrosis, inflammation, and vascular tone. Over 60 phytocannabinoids have been isolated from *Cannabis sativa* L. (family Cannabaceae). Considering the association of CB1 receptor ligands with significant psychiatric adverse effects, research efforts have increasingly focused on the development of synthetic CB2-selective cannabinoids (e.g., 5-(tert-Butyl)-N-(1-hydroxy-2-methylpropan-2-yl)-1-(5-(trifluoromethyl)pyridin-2-yl)-1H-pyrazole-3-carboxamide, WIN55212-2) [46]. A selective CB2 receptor agonist has been shown to exert antifibrotic effects by attenuating leukocyte infiltration in the skin and limiting fibrosis-associated tissue injury [130].

Ciplukan herb (*Physalis angulata* L., family Solanaceae) ethanol extract contains higher phenol concentration compared to methanol or hexane extracts [131]. These phenolic compounds exert antioxidant and immunomodulatory effects, protecting the lymphocytes from ROS-induced damage, and influencing the autoimmune process [132,133], while also promoting leukocyte proliferation [50].

Comparison of the two target lists—the evidence-based molecular targets identified through the literature search and the predicted targets generated using the SwissTargetPrediction tool—revealed that they are almost entirely disjunct, with the MAPK p38α representing the sole exception. This near-complete separation is likely attributable to our selection criteria, as only predicted targets with a probability greater than 0.5 were included in the analysis. Additional predicted targets with lower probabilities (less than 0.5) exist, some of which may correspond to targets already identified in the evidence-based list (e.g., MMP-1, IL-6, and MAPK ERK1/2).

Given that the pathogenesis of SSc remains only partially elucidated and highly complex, the specialized literature cannot definitively establish the role of certain molecules in its pathogenic mechanisms. In this context, Table 8 includes those molecular targets for which subsequent searches in PubMed and ScienceDirect identified studies that demonstrate or suggest a possible connection to the pathogenesis of SSc. Thus, Table 8 brings together several categories of research, such as studies that highlight the existence of a genetic susceptibility to SSc (tyrosine-protein kinase receptor FLT3 [102], P-glycoprotein 1 [93]); studies demonstrating the presence of autoantibodies such as anti-carbonic anhydrase I [68] and II [68,69], anti-DNA topoisomerase II alpha [77], or anti-estrogen receptor alpha [79]; research showing differential expression levels of certain molecules in SSc (apoptosis regulator Bcl-2 [60] and matrix metalloproteinase-12 [87]); studies indicating a potential therapeutic benefits of inhibitors targeting certain molecules (e.g., acetylcholinesterase [56,57 and aldolase reductase [59]); and studies reporting altered values of certain molecules (e.g., matrix metalloproteinase-12 [87] and matrix metalloproteinase-13 [88]).

The extent to which these targets contribute to the various branches of the pathogenic triad of SSc is more or less clear. For instance, aldose reductase, a phase I metabolizing enzyme, was recently suggested to be a potential major player in skin fibrosis [59]. An overactive aldose reductase, by catalyzing NADPH-dependent detoxification of various substrates, may lead to NADPH depletion, increased oxidative stress, which further triggers inflammatory signaling pathways and fibrosis [134]. Despite the fact that tissue activity of aldose reductase has not yet investigated in humans with SSc, its inhibition has proved a skin antifibrotic effect in vivo model. Similarly, MMP-12 and MMP-13 represent well-documented contributors to the fibrotic component of SSc pathogenesis. These enzymes, through their role in extracellular matrix remodeling, have been implicated in fibrotic tissue involvement [87,88]. Thus, it would be worthwhile to evaluate the anti-SSc therapeutic relevance of the phytochemicals that exert inhibitory activity on these targets.

With respect to the inflammatory branch of the pathogenic triad in SSc, Toll-like receptors TLR7 and TLR9 have been identified as key, but functionally opposing regulators of immune dysregulation [101]. In a murine model of SSc, deletion of TLR7 attenuated skin and lung fibrosis and was associated with reduced infiltration of pro-inflammatory and profibrotic immune cells and cytokines in the skin [101]. In contrast, deletion of TLR9 exacerbated skin and lung fibrosis and increased inflammatory cell infiltration, suggesting a protective role for TLR9 in this model [101]. These divergent effects are mediated by plasmacytoid dendritic cells and TLR-dependent induction of type I interferons, a central pathway in SSc pathogenesis [101]. Thus, phytochemicals displaying antagonist activity on TLR7 or/and/or agonist activity on TLR9 may be added to the list of anti-SSc candidate agents.

In SSc dermal fibroblasts, upregulation of NADPH oxidase 4 (NOX4) drives a sustained increase in ROS production, establishing an ROS-mediated positive feedback loop that promotes fibroblast activation, extracellular matrix synthesis, and DNA damage [90]. Oxidative stress driven by excessive ROS production has been implicated in endothelial damage and vascular remodeling, contributing significantly to the characteristic vasculopathy of SSc [135]. Thus, NOX4 appears to be involved in both the fibrotic and vascular damage pathways underlying SSc pathogenesis. Therefore, it would be reasonable to assume that the phytocompounds, which have an inhibitory interaction with NOX4, could exert some beneficial effects on both the vascular and fibrotic pathogenic branches of SSc.

## 5. Limitations of the Study

This review has several limitations that should be acknowledged. One limitation of this review is that its protocol was not prospectively registered in PROSPERO. Although a narrative synthesis with an emphasis on molecular mechanism was appropriate for addressing the research objectives, the absence of prior registration may reduce methodological transparency and increase the possibility of selective reporting. Efforts were made to mitigate this risk through a comprehensive search strategy and detailed reporting of the review methods.

The body of evidence synthesized in this review spans a broad and heterogeneous spectrum of experimental models and clinical contexts. Most available data derive from preclinical studies, including in vitro experiments and murine models of SSc, which primarily provide mechanistic insights into antifibrotic, anti-inflammatory, and antioxidant pathways relevant to SSc. In contrast, human studies mainly report clinical outcome improvements, such as changes in the MRSS, frequency and severity of Raynaud phenomenon, hand mobility, or respiratory function parameters, with limited mechanistic exploration. A major limitation of the available literature is therefore the predominance of preclinical data, with relatively few clinical studies. Accordingly, findings from preclinical and human studies were discussed separately to reflect differences in evidentiary depth; preclinical observations establish biological plausibility and mechanistic rationale, while the available human data represent an initial step toward clinical translation. Overall, the current evidence should be interpreted as hypothesis-generating rather than practice-changing, underscoring the need for well-designed clinical studies.

Although the experimental evidence summarized in this review highlights multiple molecular pathways through which plant-derived compounds may modulate fibrosis, inflammation, and endothelial dysfunction in SSc, several pharmacological and clinical aspects warrant careful consideration. The majority of the available data originates from in vitro and animal models, in which the concentrations used often exceed those achievable or tolerable in humans. Consequently, issues related to bioavailability, pharmacokinetics, and dosing feasibility represent major translational barriers. For example, curcumin, a polyphenol isolated from *Curcuma longa* L., has demonstrated antifibrotic effects in vitro, while its in vivo bioavailability remains low [35,136]. Consequently, oral doses of curcumin required to achieve therapeutically relevant serum concentrations are very high (above 8 g) and generally intolerable [137]. However, studies have shown that co-administration of curcumin with piperine (an inhibitor of hepatic glucuronidation involved in curcumin metabolism) can enhance curcumin bioavailability by up to twofold [138]. Furthermore, long-term safety data for most of the reviewed compounds remain scarce. While short-term experimental studies suggest favorable toxicity profiles, chronic administration, particularly in patients with multisystem involvement, requires cautious assessment.

Regarding SwissTargetPrediction results, the probability score does not illustrate the probability for a phytochemical of being bioactive, but only the probability to behave as a ligand for a protein [13]. SwissTargetPrediction also does not distinguish the functional nature of phytochemical—protein interaction (agonism or antagonism). While conservative, the exclusion of putative targets annotated “by homology” may inadvertently eliminate biologically meaningful interactions and thus constitutes an additional limitation. Furthermore, variability in target nomenclature may have led to the omission of certain molecular targets relevant to SSc pathogenesis during the third search.

## 6. Future Direction of Research

Evidence from both in vivo (SSc animal models) and in vitro (skin biopsies from SSc patients) studies indicates that nuclear factor-erythroid 2-related factor 2 (Nrf2) activation confers both antifibrotic and anti-inflammatory benefits [139]. Therefore, it would be a reasonable approach to further investigate whether certain phytochemicals that have already displayed in other disease models interfere with the pathogenic triad of SSc, and might also have potential therapeutic benefits in SSc.

Regarding the first branch of the triad (inflammation), sulforaphane, an isothiocyanate derived from *Brassica oleracea* var. *italica Plenck* (family Brassicaceae), exhibited potent antioxidant and anti-inflammatory activities. These effects are primarily mediated through activation of the Nrf2 pathway, leading to the upregulation of antioxidant gene transcription (NADPH quinone oxidoreductase-1 and glutathione S-transferase) and the suppression of proinflammatory gene (IL-6 and IL-1β) transcription. Additionally, sulforaphane modulated the NF-kB signaling pathway by preventing the phosphorylation of IkBα, thereby restricting NF-kB nuclear translocation and subsequently suppressing iNOS (inducible form of nitric oxide synthase) expression [137].

A wide spectrum of phytochemicals, whose role was not yet fully explored in conjunction with Nrf2-SSc relationship, have also shown antioxidant potential through activation of the Nrf2 signaling pathway (carnosic acid [140], withaferin A [140], luteolin [140], salidroside [141], naringenin [141], resveratrol [141], sesaminol [141], ellagic acid [141], ginsenoside Re [141], tanshinone I [141], curcumin [141], naringin [141], tetramethylpyrazine [141], withametelin [141], magnolol [141], piperine [141], myricetin [141], parthenolide [142], vitexin [143], aspalathin [143], morin [143], silibinin [143], daphnetin [143], epigallocatechin 3-gallate [143], and quercetin [143]). Among these, flavonoids (luteolin, naringenin, naringin, myricetin, quercetin, vitexin, aspalathin, and morin) and phenolic acids (ellagic acid, resveratrol, salidroside, daphnetin, and epigallocatechin-3-gallate) constitutes the largest subgroups, highlighting the central role of polyphenols in redox modulation that could partially be explained by their ability to activate Nrf2 and regulate the transcription of antioxidant and cytoprotective genes. This shared mechanism of action underscores the therapeutic potential of naturally occurring Nrf2 activators as modulators of the oxidative pathway implicated in the pathogenesis of SSc.

Regarding the second and third branches of the triad (vascular injury and fibrosis), TGF-β-dependent hyperactivation of signal transducer and activator of transcription 3 (STAT3) represents another key mechanism of fibroblast activation that promotes the progression of fibrosis in SSc [144]. Evidence from an in vivo murine model of SSc demonstrates that STAT3 inhibition suppresses TGF-β myofibroblast differentiation, reduces collagen production and profibrotic gene expression, and improves skin fibrosis [144]. Therefore, STAT3 represents a critical molecular checkpoint in fibrosis, and its pharmacological inhibition could serve as a viable treatment option for SSc. Several phytocompounds, which have been reported to inhibit STAT3, not yet studied in connection with SSc, might have certain benefits (curcumin [145], resveratrol [146], epigallocatechin-3-gallate [147], ginsenosides [148], quercetin [149], pterostilbene [150], piperine [151], embelin [152], andrographolide [153], baicalein [154], luteolin [155], delphinidin [156], piceatannol [157], parthenolide [158], magnolol [159], and indirubin [160]).

The efficacy of all these phytochemicals has to be evaluated in SSc animal models or in vitro experiments based on fibroblasts derived from SSc patients, in order to establish their potential therapeutic relevance in SSc.

Other future research directions might be to identify potential synergisms between these phytochemicals and standard conventional therapy and to analyze their putative utility as complementary therapy in SSc.

Their extensive structural optimization may also be potentially useful in designing new drugs with clinical therapeutic efficacy in SSc.

The safety and potential long-term effects of these phytochemicals or plant extracts remain to be established, as they have not been investigated in most cases.

## 7. Conclusions

According to our search, several phytocompounds (e.g., halofunginone and tanshinone IIA) and whole plant extracts (e.g., caper bush and ciplukan) proved beneficial influences on the pathogenesis of SSc, and in some cases (e.g., proanthocyanidin and bromelain) even some degree of clinical therapeutic efficacy.

In conclusion, given the current lack of effective therapeutic strategies for SSc and the growing inclination of patients toward natural treatment options [161], the use of phytocompounds or plant-derived extracts emerges as a potentially valuable complementary or alternative approach for this pathology.

Although both in vivo and in vitro findings show encouraging outcomes, indicating that phytocompounds may exert multi-target effects on molecular pathways driving fibrosis, inflammation, and oxidative stress involved in SSc pathogenesis, a substantial gap remains in the clinical evidence.

Robust clinical trials are required to evaluate the safety and therapeutic efficacy of the identified phytocompounds in patients with SSc, with careful consideration of personalized medicine strategies given the pronounced heterogeneity of the disease.

## Figures and Tables

**Table 1:** In vitro studies with single phytocompounds.

Compund Name (Chemical Class)	Natural Source	Types of Cells	Mechanism of Action
Halofunginone (Alkaloid)	*Hydrangea febrifuga* (Lour.) Y.De Smet & Granados, family Hydrangeaceae	Fibroblasts from three cGvHD patients, one SSc patient, and one healthy subject, incubated 24 h with halofuginone (10^−7^, 10^−8^, 10^−9^, and 10^−10^ M)	- ↓ TGF-β-induced collagen synthesis in a dose-dependent manner ↓ collagen α1 (I) gene expression (AC: 10^−10^ M for normal and SSc fibroblasts, 10^−8^–10^−7^ for cGvHD-derived cells ↓ CDP production (AC: 10^−9^ M for normal and SSc fibroblasts, 10^−7^ M for cGvHD-derived cells) [18]
Crocetin (Carotenoid)	*Crocus sativus* L., family Asparagales	Fibroblasts from three SSc patients who had not received any treatment and three healthy subjects—incubated 24 h with crocetin (0.1, 1, 10 μM); Cells incubated without crocetin were used as controls	- ↓ proliferation of fibroblasts from SSc and normal patients in a dose- and time-dependent manner - ↓ procollagen α1 (I), procollagen α1 (III), and MMP-1 mRNA levels in SSc and normal fibroblast (1 μM—the most pronounced effect) - ↑ TIMP-1 mRNA levels in SSc and normal fibroblasts (AC: 0.1 and 10 μM) - ↓ αSMA expression in SSc and normal fibroblasts (1 μM—the most pronounced effect) [19]
Tanshinone IIA (Diterpenoid quinone)	*Salvia miltiorrhiza* Bunge, family Lamiaceae	DVSMCs isolated from skin biopsies of 10 SSc patients with microangiopathy—incubated with tanshinone IIA (1, 10, and 100 μg/mL) in the presence of either IL17-A (100 ng/mL) or 5% serum obtained from SSc patients or healthy controls	- ↓ DVSMC proliferation induced by IL17-A in a dose- and time-dependent manner - ↓ DVSMC collagen production mediated by IL17-A (↓collagen I and collagen III expression in the presence of IL17-A or 5% SSc serum) - ↓ DVSMC migration from media to intima in the presence of IL17-A or 5% SSc serum - ↓ IL17-A or 5% SSc serum induced ERK phosphorylation (potentially critical signaling pathway in the IL17-A-driven proliferation of DVSMCs); no effects were observed on JNK or p38 phosphorylation [20]
		HUVECs pre-treated for 2 h with Tanshinone II-A (10 μg/mL), rapamycin (200 nM), or DMSO (0.05%), followed by bleomycin (2 μg/mL) exposure, after which protein expression was assessed. HVUECs incubated for 2 h with Tanshinone II-A (5, 10, and 15 μg/mL) and bleomycin (2 μg/mL), after which morphological examination and Western blot analysis were performed.	- ↓ bleomycin-induced EndoMT ↓ bleomycin induced mesenchymal cells biomarkers (αSMA and FSP-1) expression; dose-dependent ↓ in αSMA ↑ preservation of endothelial cells biomarkers (CD31 and VE-cadherin); dose-dependent ↑ in CD31 restored bleomycin-impaired tube formation - Tanshinone II-A as effective as rapamycin in preserving the endothelial phenotype in bleomycin-treated cells [21]
Resveratrol (Non-flavonoid polyphenol)	*Vitis vinifera* L., family Vitaceae	Fibroblasts isolated from skin biopsies of 10 SSc patients and 10 healthy controls treated with bleomycin (100 mmol/mL) for 24 h. Fibroblasts of SSc patients distributed in three groups: control (no treatment), bleomycin (100 mmol/mL), and bleomycin plus resveratrol (5, 10, and 20 μM).	- ↑ SIRT1 level (10 μM—the most pronounced effect) - ↓ bleomycin induced mTOR level (10 μM—the most pronounced effect) - ↓ bleomycin induced upregulation of fibrotic genes (Collagen α1 (I), Collagen α2 (I), and αSMA) - ↓ bleomycin induced upregulation of pro-inflammatory cytokines (IL-1β and IL-6) [22]
Magnesium lithospermate (Phenolic acid)	*Salvia miltiorrhiza* Bunge, family Lamiaceae	Fibroblasts isolated from skin biopsies of healthy subjects treated for 48 h with magnesium lithospermate (1, 10, 50, 100, and 150 μg/mL) and [^3^H] proline for the last 18 h.	- ↓ collagen synthesis in a dose-dependent manner - ↓ prolyl and lysyl hydroxylases (AC: 50 μg/mL) - ↓ lysyl hydroxylase, with no effect on prolyl hydroxylase (AC: 5 μg/mL) - iron (50 μM) fully reversed inhibition at 5 μg/mL; partial reversal at 50 μg/mL [23]
Dipropyltetrasulfide (organosulfur)	*Allium* L., family Amaryllidaceae	Fibroblasts isolated from skin biopsies of six-week-old female mice received daily intradermal injections for 6 weeks with 300 μL of either HOCl-generating solution or PBS (control group). Levels of H_2_O_2_ and GSH were assessed after 48 h of fibroblast incubation with dipropyltetrasulfide (2.5, 5, 10, 20, 40 μM) or medium alone (control group). Viability and cell proliferation were evaluated following a 48 h incubation of fibroblasts with dipropyltetrasulfide (10, 20, 30, 40 μM), during which [^3^H] thymidine (1 μCi/well) was administered in the final 18 h. Apoptosis and necrosis were assessed after 5, 10, 15, or 24 h incubation with dipropyltetrasulfide (10, 20, 40 μM).	- ↓ fibroblasts proliferation with greater effect on HOCl fibroblasts than on normal cells - prooxidant effect in HOCl fibroblasts: ↑ H_2_O_2_ production in a dose-dependent manner, and ↓ GSH level at all doses - ↑ cytotoxic effects on HOCl fibroblasts through an apoptotic process (40 μM—the most pronounced effect) [24]
Geniposide (iridoid glycoside)	*Gradenia jasminoides* J.Ellis, family Rubiaceae	HUVECs were randomized into four treatment groups: control (PBS), bleomycin control (bleomycin 0.1 mU/mL), bleomycin + geniposide (bleomycin 0.1 mU/mL + geniposide 200 μg/mL), and bleomycin + rapamycin (bleomycin 0.1 mU/mL + rapamycin 100 nM). Cells were harvested 48 h after treatment for mRNA and protein expression analysis.	- ↓ bleomycin-induced EndoMT (via ↓ mTOR signaling pathway): ↓ phospho-mTOR and phospho-S6) expression ↓ mesenchymal cells biomarkers (αSMA and FSP-1) expression ↑ endothelial cell biomarkers (E-cadherin and CD31) expression ↑ tube formation ↓ EndoMT key factors (Slug, Snail, and Twist) expression [25]
Abscisic acid (sesquiterpenoid) [26]	*Pinus densiflora* Siebold & Zucc., family Pinaceae [27]	Fibroblast isolated from skin biopsies of 11 SSc patients and 9 healthy controls. Cell migration was evaluated after 15 h incubation with abscisic acid (10 μM) or TGF-β (10 ng/mL). Cell proliferation was assessed after 7 days of incubation with abscisic acid (10 μM) or FGF (10 ng/mL). Evaluation of collagen type I, MMP-1 activity, and TIMP-1 expression was evaluated after 24 h incubation with or without abscisic acid (10 μM).	- Abscisic acid plasma levels were lower in SSc patients compared to controls - ↓ migration of SSc fibroblasts - ↑ proliferation of SSc fibroblasts with no effect on normal fibroblasts - ↓ collagen level in SSc fibroblasts with no effect on normal fibroblasts - ↑ MMP-1 activity in SSc fibroblasts - ↓ TIMP-1 levels in SSc fibroblasts - UV-B irradiation ↑ abscisic acid level in SSc fibroblasts [28]
HSc025—a derivate from trihydroxy-α-sanshool (amide alkaloid) [29]	*Zanthoxylum piperitum* bungeanum Maxim., family Rutaceae	Healthy human fibroblasts were incubated for 1 h with HSc025 at various concentrations and after that for 30 h with or without TGF-β (5 ng/mL). After that the luciferase and β-galactosidase were assessed. Fibroblasts were incubated for 36 h with HSc025 and small interfering RNA (2 μg ) to evaluate YB-1.	- ↓ transcription of collagen α2 (I) gene via transient activation of YB-1 translocation into the nucleus - ↓ -342COL-Luc and -161COL-Luc activities; - ↑ YB-1 nuclear level and ↓ YB-1 cytoplasmic level - ↓ TGF-β-stimulated collagen expression - ↓ TGF-β-induced -342COL-Luc activity - ↓ TGF-β-induced SBE_4_-Luc activity in a dose-dependent manner - ↓ TGF-β-induced collagen α2 (I) gene and fibronectin mRNA levels result in ↓ collagen level (evaluated by hydroxyproline level) - ↓ TGF-β/Smad3 pathway via YB-1 translocation into the nucleus [30]
Kaempferol (flavonoid)	Tea, broccoli, apples, strawberries, beans	Human skin fibroblasts from three patients with diffuse cutaneous type of SSc and three healthy subjects, incubated with or without 1 mM H_2_O_2_, with or without kaempferol for 2 h	- antiapoptotic effect ↓ number of TUNEL^+^ apoptotic cells ↓ number of apoptotic cells in a dose-dependent manner - antioxidant effect: ↓ H_2_O_2_-induced ROS accumulation ↓ NOX2 expression [31]
Celastrol (pentacyclic triterpenoid)	*Tripterygium wilfordii* Hook.f., family Celastrales	Raw 264.7 cells and bone marrow-derived macrophages were incubated with celastrol (1 μg, 5 μg) for 10 min and then treated with lipopolysaccharide (100 ng/mL) for 12 h.	- anti-inflammatory effect: ↓ mRNA expression of pro-inflammatory cytokines (TNF-α, IL1β, CXCL10, and iNOS) [32]
Epigallocatechin-3-gallate (polyphenol)	*Camellia sinensis* (L.) Kuntze, family Theaceae	Skin fibroblasts from eight SSc patients who had not received immunosuppressive treatment and eight healthy controls. Human dermal fibroblast cell line AG1518 from fetal foreskin was also used. Cells were incubated for 0–48 h with epigallocatechin-3-gallate (1–100 μM) with or without TGF-β (10 ng/mL) or PDGF-BB (20 ng/mL). Additionally, treatments include other antioxidants: SOD (150 U/mL), catalase (300 U/mL), NAC (1–10 mM), and DPI (1–20 μM). ROS stimulants -H_2_O_2_ (500 μM), SIN-1 (500 μM)-, preincubation 1 h with ERK kinase inhibitor U0126 (10 μM) or epigallocatechin-3-gallate before TGF-β/PDGF-BB were used as controls.	- antifibrotic effect: ↓ collagen type I and fibronectin in the medium of SSc fibroblasts after 24 h (AC: 40 μM) ↓ intracellular collagen type I expression in SSc fibroblasts after 24 h (AC: 40 μM) ↓ TGF-β induced expression of collagen type I and fibrotic marker CTGF in SSc fibroblasts (AC: 40 μM) no effect was observed on αSMA expression ↓ basal and TGF-β induced gel contraction in SSc fibroblasts (AC: 40 μM) - antioxidant effect: ↓ ROS production in TGF-β, H_2_O_2_, and SIN-1 stimulated SSc fibroblasts (AC: 100 μM) - ↓ MAPK pathway ↓ phospho-ERK 1/2 after 24 h in SSc fibroblasts (AC: 40 μM) ↓ PDGF-BB-induced phospho-ERK 1/2 after 1 h (AC: 40 μM) - ↓ NF-kB p65 DNA binding activity [33]
Dihydromyricetin (flavonoid)	*Nekemias grossedentata* (Hand.-Mazz.) J.Wen & Z.L.Nie, family Vitaceae	Helper T cells activated by application of anti-mouse CD3 and anti-mouse CD28 antibodies and subsequently incubated overnight with various doses of dihydromyricetin (5,10, and 20 μM/mL). After that, a stimulation cocktail was added and incubated for an additional 4 h.	- ↓ percentage of activated T cells in a dose-dependent manner - ↓ production of IL-17A and INFγ [34]
Curcumin (polyphenol) [35]	*Curcuma longa* L., family Zingiberaceae	Fibroblasts obtained from skin biopsies of five SSc immunosuppressant naive patients treated with curcumin (2, 5, 10 μM) prior to incubation with TGF-β (5 ng/mL).	- antifibrotic effect: ↓ TGF-β-induced expression of profibrotic genes (type I collagen, fibronectin, and PAI-1) in a dose-dependent manner ↓ TGF-β-Smad2/3 signaling pathway (↓ Smad2 phosphorylation and ↑ TGIF level via ↓ of proteasome-mediated degradation) [36]
Ursolic acid (pentacyclic triterpene)	Bushen Yijing Traditional Chinese Polyherbal Formula (*Astragalus membranaceus*, *Poria cocos*, *Rehmannia glutinosa* Steud., *Donkey-hide gelatin*, *Fructus Corni*, *Rhizoma Dioscoreae*, and *Carthamus tinctorius*)	HUVECs pretreated with TGF-β1 (10 ng/mL) were treated with ursolic acid and Bushen Yijing Compound containing serum	- ↓ EndoMT (via↓aKT/mTOR pathway): ↓ Snail, Slug, and Twist mRNA and protein levels ↓ aKT and mTOR phosphorylation ↓ mesenchymal cells biomarkers (αSMA and Vimentin) ↑ endothelial cells biomarkers (CD31 and E-cadherin) - ↓ TGF-β1 induced angiogenesis inhibition [37]
Verbascoside and isoverbascoside (phenylethanoid glycosides)	*Cistanche phelypaea* (L.) Cout., family Orobanchaceae	Murine normal lung fibroblast cell line MLg 2908 and human primary pulmonary cells pretreated with different doses of Nintedanib, Pirfenidone, verbascoside, or isoverbascoside were treated with TGF-β1.	- antioxidant effect: ↓ intracellular ROS levels - antifibrotic effect via ↓ Smad and non-Smad pathways (verbascoside had no effect in lung fibroblasts): ↓ collagen I levels ↓ Smad2/3 phosphorylation ↓ ERK1/2 and p38 MAPK phosphorylation [38]

Legend. ↓—reduction/inhibition; ↑—increase/activation; AC—active concentration; SSc—systemic sclerosis; cGvHD—chronic graft-versus-host disease; TGF-β—transforming growth factor β; CDP—collagenase digestible proteins; MMP-1—matrix metalloproteinase-1; mRNA—messenger ribonucleic acid; TIMP-1—tissue inhibitor of matrix metalloproteinase-1; αSMA—alpha smooth muscle actin; DVSMCs—dermal vascular smooth muscle cells; ERK—extracellular signal-regulated kinase; JNK—Jun N-terminal kinase; HUVECs—human umbilical vein endothelial cells; DMSO—dimethylsulfoxide; EndoMT—endothelial to mesenchymal transition; FSP-1—fibroblast-specific protein 1; SIRT1—silent information regulator 1; mTOR—mammalian target of rapamycin; GSH—reduced glutathione; FGF—fibroblast growth factor; YB-1—Y-box binding protein; NOX2—NADPH oxidase 2; CB2—cannabinoid receptor type 2; CXCL10—CXC motif chemokine ligand 10; TNF-α—tumor necrosis factor α; iNOS—inductible form of nitric oxide synthase; PDGF-BB—platelet-derived growth factor-BB; SOD—superoxide dismutase; NAC—N-acetyl-cysteine; ROS—reactive oxygen species; H_2_O_2_—hydrogen peroxide; SIN-1—sydnonimine-1; CTGF—connective tissue growth factor; PAI-1—plasminogen activator inhibitor-1; TGIF—TGF-β-induced factor; UV-B—ultraviolet B radiation; HOCl—hypochlorous acid; PBS—phosphate-buffered saline; DPI—diphenyleneiodonium; MAPK—mitogen-activated protein kinase; INFγ—interferon gamma; NF-kB—nuclear factor kappa-light-chain-enhancer of activated B cells; IL-1β—interleukine 1β; IL-6—interleukine 6; IL17-A—interleukine 17-A.

**Table 2:** Human studies with single phytocompounds.

Compund Name (Class of Compound)	Natural Source	Subjects	Mechanism of Action and Observed Clinical Outcomes
Proanthocyanidin (flavonoid)	*Vitis vinifera* L., family Vitaceae	SSc patients who had not received any antioxidant treatment in the past 15 days, randomized into two groups: a control group and a group treated for 30 days with Activin (100 mg/kg/day), a proanthocyanidin extract (75–80% oligomeric proanthocyanidins and 3–5% monomeric proanthocyanidins).	- ↓ adhesion molecules (ICAM-1, VCAM-1, and E-selectin) - antioxidant effect: ↓ reperfusion generated oxidative stress (↓ MDA level) [39]
Bromelain (proteolytic enzyme)	*Ananas comosus* (L.) Merr., family Bromeliaceae	A 32-year-old patient with SSc received daily Bromelain for 3 months	- ↑ hand mobility (closure of hand improved by 85%) - improve dysphagia - improve depigmented lesions of the forehead and scalp [40]

Legend. ↓—reduction/inhibition; ↑—increase/activation; SSc—systemic sclerosis; ICAM-1—intracellular adhesion molecule 1; VCAM-1—vascular cell adhesion molecule 1; MDA—malondialdehyde.

**Table 3:** In vivo studies with single phytocompounds.

Compund Name (Class of Compound)	Natural Source	Animal Model	Mechanism of Action
Crocetin (Carotenoid)	*Crocus sativus* L., family Asparagales	Six-week-old female mice, sc injected on the shaved dorsal skin with 100 μL of either bleomycin (0.2 g/L) or PBS (0.01 M) once daily for 21 consecutive days. Simultaneously, the mice from the bleomycin group received daily for 14 consecutive days ip injections of either crocetin (50 mg/kg/day) or CMC (0.5%). Blood samples were collected at the end of weeks 1, 2, 3, 5, and 7.	- ↓ procollagen α1 (I) mRNA level in the dorsal skin and lungs, particularly during the early stage (weeks 1–3) - ↓ plasma ET-1 levels only at the end of week 1 - ↓ ET-1 mRNA levels in the skin and lungs only at the end of week 1 - ↓ skin thickening (assessed between the epidermal-dermal junction and dermal-fat junction) - ↓ the percentage of lung tissue fibrosis [19]
Tanshinone II-A (Diterpenoid quinone)	*Salvia miltiorrhiza* Bunge, family Lamiaceae	48 seven-week-old female mice, sc injected daily for 21 days with 100 μL/day of either bleomycin (500 μg/mL—assigned to the bleomycin, Tanshinone II-A, and rapamycin groups—or saline—in the control group. In parallel, Tanshinone II-A (10 mg/kg/day), rapamycin (1.5 mg/kh/day), or vehicle control was administered ip. Skin biopsies were collected on day 21.	- ↓ skin thickening (assessed between the epidermal-dermal junction and dermal-fat junction) - ↑ preservation of dermal white adipose tissue (attenuation of bleomycin-induced loss) - ↓ skin extracellular-matrix (collagen content) via ↓ collagen α1 (I) and collagen α2 (I) mRNA expression in the skin - ↓ level of EndoMT: ↓ proportion of CD31/αSMA cells in the dermis ↓ bleomycin-induced Slug and Twist expression ↓ number of Slug-immunopositive endothelial cells ↓ Akt/mTOR/p70S6K pathway [21]
Magnesium lithospermate (Phenolic acid)	*Salvia miltiorrhiza* Bunge, family Lamiaceae	Six 10-week-old male hairless mice divided into two groups: treatment group—magnesium lithospermate (1 mg/kg/day) orally for 30 days and control group. On the last day [2,3-^3^H] proline (200 μCi/50 g body weight; 53 Ci/mmol) was administered ip. Dorsal skin was harvested for analysis.	- ↑ collagen synthesis - ↓ prolyl hydroxylase - ↑ collagen solubility (underhydroxylated collagen) [23]
Dipropyltetrasulfide (organosulfur)	*Allium* L., family Amaryllidaceae	Six-week-old female mice received daily intradermal injections for 6 weeks with 300 μL of either HOCl-generating solution or PBS (control group). Additionally, they were divided into groups that either received dipropyltetrasulfide treatment or did not. Biological samples collected at the end of week 7.	- ↓ skin thickening in HOCl mice, confirmed by histopathological analysis of skin biopsies - ↓ HOCl induced accumulation of collagen (I) in the skin and lung (↓ skin and lung fibrosis) - ↓ pSmad2/3 and αSMA expression in HOCl mice - ↓ advanced oxidation protein products in HOCl mice - ↓ anti-DNA-topoisomerase 1 antibodies in HOCl mice - ↓ number of splenic B cells in HOCl mice - ↓ proliferation rate of T and B cells after stimulation with anti-CD3 antibodies and lipopolysaccharide, respectively, in HOCl mice - ↓ IL-4 and IL-13 levels in HOCl mice [24]
Nimbolide (triterpene)	*Azadirachta indica* A.Juss., family Meliaceae	7–-week-old male mice were randomized into four groups and received daily treatment for 28 days: normal control (saline sc), bleomycin control (bleomycin 50 μg sc), bleomycin + nimbolide low dose (bleomycin 50 μg sc + nimbolide 1 mg/kg ip), and bleomycin + nimbolide high dose (bleomycin 50 μg sc + nimbolide 3 mg/kg ip). Skin biopsies were collected from the bleomycin injection sites.	- antioxidant effect: ↓ NO level (AC: 3 mg/kg) -anti-inflammatory effect: ↓ TNF-α, IL-1β, and pNF-kB levels in a dose-dependent manner and ↓ IL-6 expression (AC: 3 mg/kg) - antifibrotic effect: ↓ skin thickness in a dose-dependent manner ↓ disruption of dermal architecture in a dose-dependent manner ↓ extracellular matrix deposition (evaluated by collagen expression) in a dose-dependent manner ↓ Collagen α2 (I) and Collagen α1 (III) expression in a dose-dependent manner ↓ TGF-β1 and pSmad2/3 expression in a dose-dependent manner ↓ LOXL2 expression in a dose-dependent manner - ↓level of EndoMT: ↓ mesenchymal cells biomarkers (αSMA and N-cadherin) expression in a dose-dependent manner [41]
Genipsoide (iridoid glycoside)	*Gradenia jasminoides* J.Ellis, family Rubiaceae	Female mice randomized into four groups and received daily treatment for four weeks: control (PBS 100 μL sc), bleomycin control (bleomycin 100 μg sc), bleomycin + geniposide (bleomycin 100 μg sc + geniposide 40 mg/kg/day), and bleomycin + rapamycin (bleomycin 100 μg sc + rapamycin 1.5 mg/kg/day). Skin biopsies were collected from the bleomycin injection sites.	- ↓ bleomycin-induced capillary loss - ↓ bleomycin-induced fibrosis - ↓ level of EndoMT: ↓ αSMA-positive cells ↓ repressors of E-cadherin (Slug and Snail) expression ↓ number of Snail-positive endothelial cells [25]
HSc025—a compound derived from trihydroxy-α-sanshool (amide alkaloid) [29]	*Zanthoxylum piperitum* bungeanum Maxim., family Rutaceae	Four-week-old female tight skin mice randomized into three groups and treated for 14 days as follows: halofunginone (1 μg/day ip), HSc025 (15 mg/kg/day orally), and an untreated group (control). Skin biopsies were collected for hypodermal thickness measurement. Seven-week-old male mice received one dose of bleomycin (8 mg/kg intratracheal). At the same time 14 days of treatment with halofuginone (1 μg/day ip) or HSc025 (15 mg/kg/day orally) was started.	- Skin antifibrotic effect was observed for both halofunginone and HSc025 (the latter exhibited less effective): ↓ hypodermal thickness ↓ hydroxyproline level ↓ collagen level ↓ number of α-SMA-positive myofibroblasts - Pulmonary antifibrotic effect was observed for HSc025: ↓ Ashcroft score of fibrosis ↓ Hydroxyproline level ↓ number of myofibroblasts [30]
Withaferin A (steroidal lactone)	*Withania somnifera* (L.) Dunal, family Solanaceae	Eight-nine-week-old male mice were randomized into five groups and received daily treatment for 28 days: normal control (saline sc), bleomycin control (bleomycin 50 μg sc), bleomycin + withaferin A low dose (bleomycin 50 μg sc + withaferin A 2 mg/kg ip), bleomycin + withaferin A high dose (bleomycin 50 μg sc + withaferin A 4 mg/kg ip) and withaferin A control (withaferin A 4 mg/kg). Skin biopsies were collected from the bleomycin injection sites. Skin tissue supernatants were used for future investigations.	- ↓ skin thickening - antioxidant effect ↑ GSH level in a dose-dependent manner ↓ NO level in a dose-dependent manner ↓ iNOS expression in a dose-dependent manner - ↓ bleomycin-induced EndoMT ↑ endothelial cells biomarker (E-Cadherin) expression in a dose-dependent manner ↓ mesenchymal cells biomarkers (αSMA, fibronectin, and vimentin) expression in a dose-dependent manner - restored dermal architecture in a dose-dependent manner ↓ accumulation of collagen ↓ hydroxyproline level ↓ Collagen α2 (I) and Collagen α1 (III) expression - ↓ TGF-β/Smad pathway ↓ TGF-β1 expression in a dose-dependent manner ↓ pSmad2/3 in a dose-dependent manner - ↓ FoxO3a/Akt/NF-kB/IKK cascade ↓ pAkt expression in a dose-dependent manner ↓ p-NF-kB and p-IKKβ expression in a dose-dependent manner ↓ BRD4 expression in a dose-dependent manner ↓ p-p38 MAPK and p-p44/42 MAPK expression in a dose-dependent manner ↑ FoxO3a expression in a dose-dependent manner - ↓ TNF-α and IL-1β expression [42]
Kaempferol (flavonoid)	Tea, broccoli, apples, strawberries, beans	Eight-week-old mice received injections of 300 μL bleomycin (1 mg/mL), five times per week for a duration of two weeks. They also received ip injections with kaempferol (40 mg/mL) or DMSO, five times per week for a duration of two weeks. Skin biopsies were collected from the bleomycin injection sites.	- ↓ Bleomycin-induced skin thickening - antifibrotic effect ↓ Bleomycin-induced collagen deposition ↓ Bleomycin-induced connective-tissue growth factor expression ↓ number of αSMA-positive myofibroblasts - antioxidant effect ↓ HO-1 and NOX2 mRNA expression No effect on Trx2 mRNA expression was observed - ↓ number of inflammatory cells (CD3^+^ T-cells and CD68^+^ macrophages) in the dermis - ↓ mRNA expression of inflammatory and pro-fibrotic cytokines (IL-6, TNF-α, and TGF-β) - ↓ caspase-3 activity, which is an apoptosis marker [31]
Celastrol (pentacyclic triterpenoid)	*Tripterygium wilfordii* Hook.f., family Celastrales	Six-eight-old female mice were randomized into four groups and received daily treatment for four weeks: control (PBS 100 μg sc), bleomycin control (bleomycin 100 μg sc), bleomycin + Celastrol (bleomycin 100 μg sc + Celastrol 1 mg/kg sc), and bleomycin + WIN55212-2 (bleomycin 1000 μg sc + WIN55212-2 1 mg/kg SC). Skin biopsies were collected from the bleomycin injection sites.	- Celastrol is a selective agonist of CB2 - ↓ Bleomycin-induced skin thickening (↓ thickening of dermis) - ↓ Bleomycin-induced collagen deposition - ↓ expression of αSMA - ↓ mRNA expression of TNF-α, TGF-β, Collagen α (I), and αSMA [32]
Dihydromyricetin (flavonoid)	*Nekemias grossedentata* (Hand.-Mazz.) J.Wen & Z.L.Nie, family Vitaceae	Eight-week-old mice received daily sc injections of bleomycin (100 μg/100 μL) for 21 days. From day 10 dihydromyricetin (5 mg/kg or 10 mg/kg) was administered orally once daily. Skin biopsies were collected from the bleomycin injection sites.	- ↓ Localized Scleroderma Skin Severity Index - ↓ thickness of the dermis - ↓ antigen-presenting macrophages - ↓ percentage of M2 type macrophages and of IL-4 and IL-6 producing macrophages in a dose dependent manner - ↓ percentage of IL-10, IL-12 and IL-17 producing myeloid-derived adjuster cells - ↓ percentage of total helper T cells, cytotoxic T cells, and activated T cells - ↓ percentage of IL-4 and IL-17A producing T cells - ↓ expression of RORγt (a key factor for IL-17A production) [34]
Astragalaus polysaccharides	*Astragalus membranaceus* Fisch. Ex Bunge, family Fabaceae	Six-week-old mice received daily sc injections of 100 μL bleomycin (600 μg/mL) for 21 days or 100 μL PBS (control group). A part of the bleomycin group received *Astragalus* polysaccharides (200 mg/kg). At the end of weeks 1, 2, and 3, blood samples and skin biopsies from the bleomycin injection sites were collected.	- antifibrotic effect (via ↓ TGF-β/Smad signaling pathway): ↓TGF-β, Smad2 and Smad3 expression ↓ dermal skin thickness ↓ collagen type I level in the dermis ↓ hydroxyproline level in the dermis ↓ MCP-1 mRNA level [43]
Madecassoside (triterpenoid saponin)	*Centella asiatica* (L.) Urb., family Apiaceae	Female mice randomized into six treatment groups: control, induced pulmonary fibrosis (5 mg/kg bleomycin endotracheal), Prednisone (5 mg/kg), and Madecassoside (10, 20, and 40 mg/kg). After 21 days pulmonary tissue and bronchoalveolar lavage were collected.	- pulmonary antifibrotic effect (via ↓TGF-β/Smad pathway) ↓TGF-β1, pSmad2/3) ↓structural destruction ↓collagen deposition (↓hydroxyproline level) ↓infiltration of inflammatory cells ↓fibroblast proliferation ↓myofibroblast number (↓α-SMA level) ↑MMP1/TIMP1 mRNA ratio - antioxidant effect ↑ Cu-Zn SOD activity ↑ GSH level ↑ MDA level ↓ MPO activity - anti-inflammatory effect ↓ leukocytes number in bronchoalveolar lavage [44]
Asiaticoside (triterpenoid saponin)	*Centella asiatica* (L.) Urb., family Apiaceae	45 wild-type mice and 45 Adenosine 2A receptor gene knockout mice were divided into three groups: control, bleomycin (50 U/kg intratracheal), and bleomycin (50 U/kg intratracheal) plus asiaticoside (50 mg/kg/day gastric perfusion). After 28 days lung tissue was collected.	- pulmonary anti-inflammatory effect: ↓ alveolar inflammation scores - pulmonary antifibrotic effect (via activating cAMP/Rap1 pathway assisted by adenosine 2A receptor): ↓ collagen deposition ↓ alveolar structural damage [45]
5-(tert-Butyl)-N-(1-hydroxy-2-methylpropan-2-yl)-1-(5- (trifluoromethyl)pyridin-2-yl)-1H-pyrazole-3-carboxamide (compound 66—synthetic CB2 receptor agonist)	*Cannabis sativa* L., family Cannabaceae	Mice injected sc with bleomycin for two weeks were treated for another 4 weeks with compound 66 (1 or 5 mg/kg/day) or with compound 9—WIN55212-2 (1 mg/kg/day)	- antifibrotic effect ↓ dermal thickness ↓ α-SMA expression ↓ mRNA expression of profibrotic and proinflammatory mediators (Collagen α (I), α-SMA, TNF-α, and TGF-β) [46]

Legend. ↓—reduction/inhibition; ↑—increase/activation; AC—active concentration; SSc—systemic sclerosis; PBS—phosphate-buffered saline; CMC—carboxymethylcellulose; ET-1—plasma endothelin-1; ip—intraperitoneal; sc—subcutaneous; EndoMT—endothelial to mesenchymal transition; mTOR—mammalian target of rapamycin; HOCl—hypochlorous acid; αSMA—alpha smooth muscle actin; pSmad2/3—phosphorylated SMAD family member 2 and SMAD family member 3; NO—nitric oxide; TNF-α—tumor necrosis factor α; pNF-kB—phosphorylated nuclear factor kappa B; TGF-β1—transforming growth factor β1; LOXL2—lysyl oxidase-like protein 2; ICAM-1—intracellular adhesion molecule 1; VCAM-1—vascular cell adhesion molecule 1; iNOS—inductible form of nitric oxide synthase; FoxO3a—forkhead box O3; Akt—protein kinase B; pAkt—phosphorylated protein kinase B; NF-kB—nuclear factor kappa B; p-NF-kB p65—phosphorylated nuclear factor kappa-light-chain-enhancer of activated B cells subunit p 65; IKK—IkB kinase; p-IKKβ—phosphorylated IkB kinase β; BRD4—bromodomain-containing protein 4; p-p38 MAPK—phosphorylated p38 mitogen-activated protein kinase; p-p44/42 MAPK—phosphorylated p44-42 mitogen-activated protein kinase; DMSO—dimethylsulfoxide; HO-1—heme oxygenase-1; NOX2—NADPH oxidase 2; Trx2—thioredoxin 2; CB2—cannabinoid receptor type 2; RORγt—retinoic acid-related orphan receptor gamma t; MCP-1—monocyte chemoattractant protein-1; MMP-1—matrix metalloproteinase-1; TIMP-1—tissue inhibitor of metalloproteinases-1; MDA—malondialdehyde; SOD—superoxide dismutase; MPO—myeloperoxidase; IL-4—interleukin 4; IL-6—interleukin 6; IL-10—interleukin 10; IL-12—interleukin 12; IL-17—interleukin 17; IL-1β—interleukin 1β.

**Table 4:** In vitro studies with whole plant extracts.

Vernacular Plant Name	Latin Scientific Plant Name	Major Phytocompounds	Types of Cells	Mechanism of Action
Caper bush (ethanol extract)	*Capparis spinosa* L., family Capparidaceae	Alkaloids, lipids, polyphenols (flavonoids kaempferol, quercetin derivatives, hydroxycinnamic acids caffeic acid, p-cumaric acid, ferulic acid, and cinnamic acid), indole, and glucosinolates [47]	Fibroblasts from three SSc patients and three healthy controls treated for 48 h with ethanol extract of Capparis spinosa L. (10, 50, 100 μg/mL) or N-acetyl-L-cysteine (10 mM).	- antioxidant effect: ↓ ROS (including H_2_O_2_ and O_2_) production in SSc fibroblasts in a dose-dependent manner ↓ H_2_O_2_-induced apoptosis of SSc fibroblast in a dose-dependent manner - ↓ levels of Ha-Ras and active (phosphorylated) forms of ERK1/2, interrupting the ROS-ERK1/2-Ha-Ras loop - ↓ type I collagen level and α_2_(I) collagen mRNA gene expression in SSc fibroblast (via ROS-ERK1/2-Ha-Ras loop interruption) [48]
Caper bush (ethanol extract)	*Capparis spinosa* L., family Capparidaceae	Alkaloids, lipids, polyphenols (flavonoidskaempferol, quercetin derivatives, hydroxycinnamic acids, caffeic acid, p-cumaric acid, ferulic acid, and cinnamic acid), indole, and glucosinolates [47]	Lung fibroblasts isolated from mice assigned to several groups: control (PBS for 12 h), TGF-β1 (5 ng/mL for 12 h), Capparis spinosa low/medium/high dose (TGF-β1 5 ng/mL + Capparis spinosa 100/300/500 μg/mL for 12 h), SP600125 (1 h pre-treated with SP600125 100 μmol/L before TGF-β1 5 ng/mL) and Capparis spinosa + Anisomycin (1 h pretreated with 1 μg/mL Anisomycin followed by 12 h of TGF-β1 5 ng/mL and Capparis spinosa 500 μg/mL).	- antifibrotic effect: ↓ TGF-β1 induced myofibroblast proliferation in a dose-dependent manner ↓ TGF-β1 induced cell migration in a dose-dependent manner ↓ αSMA, collagen I, and fibronectin expression in a dose-dependent manner - ↓ MAPK pathway ↓ Bleomycin induced phosphorylation of ERK1/2, JNK, and p38 MAPK in a dose-dependent manner SP600125 (a p38 MAPH/JAK inhibitor) had similar effects to Capparis Spinosa Anisomycin (a p38 MAPK/JAK activator) reversed Capparis spinosa effects [49]

Legend. ↓—reduction/inhibition; ↑—increase/activation; SSc—scleroderma patients; ROS—reactive oxygen species; Ha—Ras—Harvey rat sarcoma viral oncogene homolog; ERK1/2—extracellular signal regulated kinase 1 and 2; PBS—phosphate-buffered saline; TGF-β1—transforming growth factor β1; αSMA—alpha smooth muscle actin; MAPK—mitogen-activated protein kinase; p38 MAPK—p38 mitogen-activated protein kinase; ERK1/2—extracellular signal-regulated Kinases 1 and 2; JNK—Jun N-terminal kinase.

**Table 5:** Human studies with whole plant extracts.

Vernacular Plant Name	Scientific Plant Name	Phytocompounds	Subjects	Observed Clinical Outcomes
Ciplukan	*Physalis angulata* L., family Solanaceae	Phenol (flavonoid, tannin, and phenylpropane), secosteroid (physalin and withanguline), and saponin	SSc patients (*n* = 59) on standard therapy with stable dose for≥3 months, randomized to treatment (Ciplukan 50% ethanol extract, 250 mg × 3/day 12 weeks, *n* = 29) or placebo (amylum 250 mg × 3/day 12 weeks, *n* = 30) groups. MRSS, ESR, BAFF, sCD40L, and P1NP were assessed every 4 weeks until the end of the study.	- ↓ MRSS after three months of treatment in ciplukan group - ↓ P1NP levels after three months of treatment in the Ciplukan group - no significant changes were observed in ESR, BAFF, or sCD40L [50]
Lei Gong Teng	*Tripterygium wilfordii* Hook.f., family Celastrales	Diterpenoids (triptolide, tripdiolide, and triptomide) and alkaloids	SSc patients diagnosed by HRCT with ILD (*n* = 76) and disease duration <seven years, treated with *Tripterygium wilfordii* Hook.f. 40–60 mg/day (*n* = 28) or cyclophosphamide 100 mg/day (*n* = 48) > 1 year. Pulmonary function test was tested ≤ 1 month before and after treatment.	- ↑ mean FVC and FVC % of predicted in the *Tripterygium wilfordii Hook*.f group only when it is administered as maintenance therapy - no statistically significant effect on FVC and FVC % in the *Tripterygium wilfordii Hook*.f induction therapy group - no statistically significant effect on TLC and DLCO - fewer adverse reactions were observed in the *Tripterygium wilfordii Hook*.f group (fewer cases of leukopenia) [51]
St. John’s Wort	*Hypericum perforatum* L., family Hypericaceae Juss.	Naphthodianthrone, flavonoids, and tannins [52]	Patients with primary Raynaud’s phenomenon and secondary Raynaud’s phenomenon associated with SSc or other connective tissue diseases treated with St. John’s Wort 300 mg twice daily or placebo for 6 weeks.	- no improvement in severity, duration, or frequency of attacks - ↑ levels of proangiogenic cytokines (MMP-9, MIP-1β, sE-selectin, G-CSF, VEGF) for patients with >50% improvement in attack frequency - ↑ levels of sVCAM-1, sICAM-1, and MCP-1 for patients with improvement of severity of attacks [53]
Evening primrose (seed oil)	*Oenothera biennis* L., family Onagraceae	Gamma-linolenic acid	21 patients with Raynaud’s phenomenon with or without SSc received either 6 g of evening primrose oil or placebo for eight weeks	- ↓number of Raynaud’s phenomenon attacks in the treatment group [54]
			25 patients with SSc received either evening primrose oil combined with fish oil or a placebo (sunflower oil and vitamin E)	- no improvement in severity, duration, or frequency of attacks [55]

Legend. ↓—reduction/inhibition; ↑—increase/activation; SSc—systemic sclerosis; MRSS—modified Rodnan skin scale; ESR—erythrocyte sedimentation rate; BAFF—B-cell activation factor; sCD40L—soluble CD40 ligand; P1NP—procollagen type I N-terminal propeptide; ILD—interstitial lung disease; FVC—forced vital capacity; TLC—total lung capacity; DLCO—diffusing capacity of the lung for carbon monoxide; MMP-9—matrix metalloproteinase-9; MIP-1β—macrophage inflammatory protein 1-β; sE-selectin—soluble E-selectin; G-CSF—granulocyte colony-stimulating factor; VEGF—vascular endothelial growth factor; sVCAM-1—soluble vascular cell adhesion molecule-1; sICAM-1—soluble intercellular adhesion molecule-1; MCP-1—monocyte chemoattractant protein-1.

**Table 6:** In vivo studies with whole plant extracts.

Vernacular Plant Name	Scientific Plant Name	Phytocompounds	Animal Model	Mechanism of Action
Caper bush (ethanol extract)	*Capparis spinosa* L., family Capparidaceae	Alkaloids, lipids, polyphenols (flavonoids kaempferol, quercetin derivatives, hydroxycinnamic acids caffeic acid, p-cumaric acid, ferulic acid, and cinnamic acid), indole, and glucosinolates [47]	Eight-week-old mice weighing 20–22 g were once daily injected sc with 0.1 mL of Bleomycin (400 μg/mL) or PBS for 28 days. Bleomycin-treated mice were randomized into four groups: Bleomycin, Capparis spinosa low dose (100 mg/kg/day), medium dose (300 mg/kg/day), or high dose (500 mg/kg/day). Capparis spinosa extract was administered by oral gavage for 4 weeks. At the end of the study, the mice were euthanized, and blood samples, BAFL, back skin ang lung biopsies were collected.	- improved mental state - anti-inflammatory effect: ↓ Infiltration of immune cells within the dermis and lung tissue ↓ TNF-α, IL-6, and IL-1β serum and BAFL levels in a dose-dependent manner - antifibrotic effect: ↓ dermis thickness ↓ collagen fiber content in the skin in a dose-dependent manner ↓ hydroxyproline contents in the skin and lung tissue in a dose-dependent manner ↓ profibrotic factors (TGF-β1 and αSMA) expression in lung tissue in a dose-dependent manner ↓ collagen I and fibronectin expression in lung tissue in a dose-dependent manner - ↓ MAPK pathway ↓ Bleomycin induced phosphorylation of ERK1/2, JNK, and p38 MAPK in a dose-dependent manner [49]

Legend. ↓—reduction/inhibition; ↑—increase/activation; SSc—systemic sclerosis; PBS—phosphate-buffered saline; BAFL—bronchoalveolar lavage fluid; TNF-α—tumor necrosis factor α; IL-1β—interleukin 1β; IL-6—interleukin 6; TGF-β1—transforming growth factor β1; αSMA—alpha smooth muscle actin; MAPK—mitogen-activated protein kinase; p38 MAPK—p38 mitogen-activated protein kinase; ERK1/2—extracellular signal-regulated Kinases 1 and 2; JNK—Jun N-terminal kinase.

**Table 7:** Summary of principal phytocompounds, molecular targets, and level of evidence.

Compound Name	Type of Study	Molecular Target
Halofunginone	In vitro	- collagen α1 (I) gene
	In vivo	- procollagen α1 (I) - ET-1
Crocetin	In vitro	- procollagen α1 (I), procollagen α1 (III), MMP-1, and TIMP-1 - αSMA
Tanshinone IIA	In vitro	- collagen I and collagen III - ERK - αSMA and FSP-1 - CD31 and VE-cadherin
	In vivo	- collagen α1 (I) and collagen α2 (I) - Slug and Twist - Akt/mTOR/p70S6K pathway
Resveratrol	In vitro	- SIRT1 - mTOR - Collagen α1 (I) and Collagen α2 (I) - αSMA - IL-1β and IL-6
Magnesium lithospermate	In vitro	- prolyl and lysyl hydroxylases
	In vivo	- prolyl hydroxylase
Dipropyltetrasulfide	In vitro	- H_2_O_2_ - GSH
	In vivo	- collagen (I) - pSmad2/3 - αSMA - anti-DNA-topoisomerase 1 antibodies - IL-4, IL-13
Geniposide	In vitro	- mTOR - αSMA and FSP-1 - E-cadherin and CD31 - Slug, Snail, and Twist
	In vivo	- αSMA - Slug and Snail
Abscisic acid	In vitro	- MMP-1 - TIMP-1
HSc025	In vitro	- collagen α2 (I) - 342COL-Luc, -161COL-Luc, and SBE_4_-Luc - collagen α2 (I) - fibronectin - TGF-β/Smad3 pathway (YB-1)
Kaempferol	In vitro	- NOX2
	In vivo	- HO-1, NOX2 - IL-6, TNF-α, and TGF-β - caspase-3
Celastrol	In vitro	- TNF-α and IL1β - CXCL10 and iNOS
	In vivo	- αSMA - Collagen α (I) - TNF-α and TGF-β
Epigallocatechin-3-gallate	In vitro	- collagen type I and fibronectin - CTGF - ROS - phospho-ERK 1/2 - NF-kB p65 - TGF-β
Dihydromyricetin	In vitro	- IL-17A and INFγ
	In vivo	- RORγt
Curcumin	In vitro	- type I collagen, fibronectin, and PAI-1 - TGF-β-Smad2/3 pathway (Smad2 and TGIF)
Ursolic acid	In vitro	- aKT/mTOR pathway - Snail, Slug, and Twist - αSMA and Vimentin - CD31 and E-cadherin
Verbascoside and isoverbascoside	In vitro	- ROS - collagen I - Smad2/3 - ERK1/2 and p38 MAPK
Proanthocyanidin	Human	- ICAM-1, VCAM-1, and E-selectin
Nimbolide	In vivo	- NO - TNF-α, IL-1β, and IL-6 - pNF-kB - TGF-β1 and pSmad2/3 - Collagen α2 (I) and Collagen α1 (III) - LOXL2 - αSMA and N-cadherin
Withaferin A	In vivo	- GSH - NO - iNOS - E-Cadherin - αSMA, fibronectin, and vimentin - Collagen α2 (I) and Collagen α1 (III) - TGF-β/Smad pathway (TGF-β1 and pSmad2/3) - FoxO3a/Akt/NF-kB/IKK cascade (pAkt, p-NF-kB, p-IKKβ, BRD4, p-p38 MAPK, p-p44/42 MAPK, and FoxO3a) - TNF-α and IL-1β
Astragalaus polysaccharides	In vivo	- TGF-β/Smad pathway (TGF-β, Smad2, and Smad3) - collagen type I - MCP-1
Madecassoside	In vivo	- TGF-β/Smad pathway (TGF-β1 and pSmad2/3) - α-SMA - MMP1/TIMP1 - Cu-Zn SOD, MPO, GSH, and MDA
Asiaticoside	In vivo	- cAMP/Rap1 pathway (adenosine 2A receptor)
5-(tert-Butyl)-N-(1-hydroxy-2-methylpropan-2-yl)-1-(5- (trifluoromethyl)pyridin-2-yl)-1H-pyrazole-3-carboxamide	In vivo	- Collagen α (I) - α-SMA - TNF-α and TGF-β

Legend: MMP-1—matrix metalloproteinase-1; TIMP-1—tissue inhibitor of matrix metalloproteinase-1; αSMA—alpha smooth muscle actin; ERK—extracellular signal-regulated kinase; FSP-1—fibroblast-specific protein 1; SIRT1—Silent information regulator 1; mTOR—mammalian target of rapamycin; GSH—reduced glutathione; YB-1—Y-box binding protein; NOX2—NADPH oxidase 2; TNF-α—tumor necrosis factor α; iNOS—inductible form of nitric oxide synthase; ROS—reactive oxygen species; H_2_O_2_—hydrogen peroxide; PAI-1—plasminogen activator inhibitor-1; TGIF—TGF-β-induced factor; MAPK—mitogen-activated protein kinase; INFγ—interferon gamma; NF-kB—nuclear factor kappa-light-chain-enhancer of activated B cells; IL-1β—interleukine 1β; IL-4—interleukin 4; IL-6—interleukine 6; IL-10—interleukin 10; IL-12—interleukin 12; IL17-A—interleukine 17-A; ET-1—plasma endothelin-1; pSmad2/3—phosphorylated SMAD family member 2 and SMAD family member 3; NO—nitric oxide; pNF-kB—phosphorylated nuclear factor kappa B; VCAM-1—vascular cell adhesion molecule 1; FoxO3a—forkhead box O3; Akt—protein kinase B; pAkt—phosphorylated protein kinase B; IKK—IkB kinase; p-IKKβ—phosphorylated IkB kinase β; BRD4—bromodomain-containing protein 4; p-p38 MAPK—phosphorylated p38 mitogen-activated protein kinase; p-p44/42 MAPK—phosphorylated p44-42 mitogen-activated protein kinase; HO-1—heme oxygenase-1; RORγt—retinoic acid-related orphan receptor gamma t; MCP-1—monocyte chemoattractant protein-1; MDA—malondialdehyde; SOD—superoxide dismutase; CTGF—connective tissue growth factor; ICAM-1—intracellular adhesion molecule 1; MPO—myeloperoxidase.

**Table 8:** Putative molecular targets of the present phytochemicals with an evidence-based role in the pathogenesis of SSc.

Molecular Target	Type of Study	References
Acetylcholinesterase	Human	[56]
	Human	[57]
Acyl coenzyme A: cholesterol acyltransferase	In vitro	[58]
Aldose reductase	In vitro + in vivo	[59]
Apoptosis regulator Bcl-2	Human + in vitro	[60]
	Human + in vitro	[61]
	Human	[62]
Arachidonate 15-lipoxygenase	In vitro + in vivo	[63]
Arachidonate 5-lipoxygenase	in vitro	[64]
	In vitro	[65]
Aryl hydrocarbon receptor	In vitro + in vivo	[66]
Beta amyloid A4 protein	In vitro + in vivo	[67]
Beta-secretase 1	Human + in vivo + in vitro	[67]
Carbonic anhydrase I	Human	[68]
Carbonic anhydrase II	Human	[68]
	Human	[69]
Carbonic anhydrase VI	Human	[70]
Carbonic anhydrase IX	Human	[71]
Cyclin-dependent kinase 5/CDK5 activator 1	In vitro + in vivo	[72]
Cyclooxygenase-1	Human	[73]
Cyclooxygenase-2	In vitro	[74]
	Human	[73]
	Human	[75]
DNA (cytosine-5)-methyltransferase 1	Human + in vivo + in vitro	[76]
DNA topoisomerase II alpha	Human	[77]
Dual-specificity phosphatase Cdc25B	Human	[78]
Estrogen receptor alpha	Human + in vitro	[79]
Glycogen synthase kinase-3 beta	In vitro + in vivo	[80]
Hepatocyte growth factor receptor	In vitro	[81]
Histone acetyltransferase p300	Human + in vivo + in vitro	[82]
MAP kinase p38 alpha	In vitro	[83]
Matrix metalloproteinase 2	Human	[84]
	Human	[85]
Matrix metalloproteinase 9	Human	[84]
	Human + in vitro	[86]
Matrix metalloproteinase 12	Human	[87]
Matrix metalloproteinase 13	Human	[88]
Matrix metalloproteinase 14	Human + in vivo + in vitro	[89]
NADPH oxidase 4	Human + in vitro	[90]
	In vivo	[91]
Nuclear receptor ROR-gamma	In vitro	[92]
P-glycoprotein 1	Human + in vitro	[93]
Protein-tyrosine phosphatase 2C	Human	[94]
PI3-kinase p110-alpha subunit	Human	[95]
Prostaglandin E synthase	In vitro	[96]
Protein kinase C alpha	In vitro	[97]
Protein-tyrosine phosphatase 1B	In vitro	[98]
Signal transducer and activator of transcription 1-alpha/beta	In vitro	[99]
Telomerase reverse transcriptase	Human	[100]
Toll-like receptor (TLR7/TLR9)	In vivo	[101]
Tyrosine-protein kinase receptor FLT3	Human	[102]
Tyrosine-protein kinase SYK	In vivo	[103]
Xanthine dehydrogenase	Human	[104]

## Data Availability

No new data were created or analyzed in this study. Data sharing is not applicable to this article.

## Supplementary Materials

The following supporting information can be downloaded at: https://www.mdpi.com/article/10.3390/cimb48010097/s1, Table S1: The main characteristics of the studies included in this systematic review; Table S2: Quality assessment of all studies evaluated using an abbreviated version of the Standard Quality Assessment Criteria for Evaluating Primary Research Papers from a Variety of Fields developed by Kmet et al.; Table S3: Risk of bias assessed using the Systematic Review Centre for Laboratory animal Experimentation (SYRCLE) Risk of Bias tool; Table S4: Risk of bias assessed using the Risk Of Bias In Non-randomized Studies—of Interventions (ROBINS-I) tool; Table S5: Evidence-based phytocompound-molecular target map in systemic sclerosis pathogenesis; Table S6: Putative molecular targets of the selected phytochemicals—identified with SwissTargetPrediction tool; Search strategy using Bollean operators; Prisma checklist.

## Abbreviations

The following abbreviations are used in this manuscript:

AC	active concentration
BAFF	B-cell activation factor
BRD4	bromodomain-containing protein 4
CB2	cannabinoid receptor type 2
CD3	cluster of differentiation 3
CD28	cluster of differentiation 28
CD31	cluster of differentiation 31
CDP	collagenase-digestible proteins
CMC	carboxymethylcellulose
cGvHD	chronic graft-versus-host disease
CTGF	connective tissue growth factor
CXCL10	CXC motif chemokine ligand 10
DLCO	diffusing capacity of the lung for carbon monoxide
DMSO	dimethylsulfoxide
DPI	diphenyleneiodonium
DVSMCs	dermal vascular smooth muscle cells
EndoMT	endothelial to mesenchymal transition
ERK	extracellular signal-regulated kinase
ESR	erythrocyte sedimentation rate
ET-1	plasma endothelin-1
FGF	fibroblast growth factor
FSP-1	fibroblast-specific protein 1
FVC	forced vital capacity
G-CSF	granulocyte colony-stimulating factor
GSH	reduced glutathione
H_2_O_2_	hydrogen peroxide
HO-1	heme oxygenase-1
HOCl	hypochlorous acid
HUVECs	human umbilical vein endothelial cells
ICAM-1	intracellular adhesion molecule 1
IKK	IκB kinase
IL-1β	interleukine 1β
IL-4	interleukine 4
IL-6	interleukine 6
IL-12	interleukine 12
IL-13	interleukine 13
IL-17A	interleukine 17A
ILD	interstitial lung disease
INFγ	interferon gamma
iNOS	inducible nitric oxide synthase
JNK	Jun N-terminal kinase
MAPK	mitogen-activated protein kinase
MCP-1	monocyte chemoattractant protein-1
MDA	malondialdehyde
MIP-1β	Macrophage inflammatory protein 1β
MMP-1	matrix metalloproteinase-1
MMP-9	matrix metalloproteinase-9
MPO	myeloperoxidase
mRNA	messenger ribonucleic acid
MRSS	modified Rodnan skin score
mTOR	mammalian target of rapamycin
NAC	N-acetyl-cysteine
NF-kB p65	nuclear factor kappa-light-chain-enhancer of activated B cells
NOX2	NADPH oxidase 2
P1NP	procollagen type I N-terminal propeptide
PAI-1	plasminogen activator inhibitor-1
PBS	phosphate-buffered saline
PDGF-BB	platelet-derived growth factor-BB
p-p44/42 MAPK	phosphorylated p44/42 MAPK
RORγt	retinoic acid-related orphan receptor gamma t
ROS	reactive oxygen species
SIRT1	silent information regulator 1
SIN-1	sydnonimine-1
SOD	superoxide dismutase
SSc	systemic sclerosis
sICAM-1	soluble intercellular adhesion molecule-1
sVCAM-1	soluble vascular cell adhesion molecule-1
TGF-β	transforming growth factor β
TGIF	TGF-β-induced factor
TIMP-1	tissue inhibitor of matrix metalloproteinase-1
TLC	total lung capacity
TNF-α	tumor necrosis factor α
Trx2	thioredoxin 2
UV-B	ultraviolet B radiation
VCAM-1	vascular cell adhesion molecule 1
VEGF	vascular endothelial growth factor
YB-1	Y-box binding protein 1
