# Supplementary material for: Plant-Derived Agents and Systemic Sclerosis: A Systematic Review of Therapeutic Potential and Molecular Mechanisms
Source: Curr Issues Mol Biol. 2026 Jan 18;48(1):97. doi: 10.3390/cimb48010097 (PMC12840015; doi:10.3390/cimb48010097)
Supplement: Supplementary file 1 [file cimb-48-00097-s001.zip › PRISMA_2020_checklist.pdf]

PRISMA 2020 Checklist

Section and Topic	Item #	Checklist item	Location where item is reported
**TITLE**			
Title	1	Plant-derived agents and systemic sclerosis: A sytematic review of therapeutic potential and molecular mechanisms	Title
**ABSTRACT**			
Abstract	2	**Background:** Systemic sclerosis (SSc) is a complex autoimmune connective tissue disease characterized by progressive fibrosis. Despite significant advances in its management over the past few decades, the disease continues to be associated with considerable morbidity and mortality. This systematic review aimed to identify phytocompounds and medicinal plants that had demonstrated efficacy in SSc.<br>**Method:** A comprehensive literature search was performed in PubMed and ScienceDirect in August 2025, with a final update in September 2025. Out of 7,797 records identified, 32 studies met the inclusion criteria. A second search was performed using SwissTargetPrediction tool to identify for these phytocompounds new putative molecular targets, whose relevance for SSc pathogenesis was verified by a third search in PubMed and ScienceDirect databases.<br>**Results:** Our search found 25 phytocompounds (e.g. halofunginone, crocetin, tanshinone IIA) and 5 plant extracts (e.g. caper bush, ciplukan) potentially useful in SSc. SwissTargetPrediction tool indicated 93 new potential molecular targets of the previously identified phytochemicals, among which only 41 showed relevance to SSc pathogenesis.<br>**Conclusions:** Available evidence is scarce, but promising. Studies specifically assessing the clinical efficacy, safety, potential interactions with drugs, and consequences of long-term use in SSc are required for most of these plant-derived agents.	Abstract
**INTRODUCTION**			
Rationale	3	According to the updated 2023 European Alliance of Associations for Rheumatology (EULAR) treatment recommendations, the management of SSc is tailored to organ-specific involvement. In Raynaud phenomenon, first line therapy consists of dihydropyridine-type calcium channel antagonists (usually Nifedipine), with escalation to phosphodiesterase-5 (PDE5) inhibitors or intravenous Iloprost in refractory cases. Digital ulcers are managed with PDE5 inhibitors, intravenous Iloprost or Bosentan. PAH requires early initiation of combination therapy with PDE5 inhibitors and endothelin receptor antagonists, additional prostacyclin analogues (e.g. Selexipag) or Riociguat where indicated, with consideration of Epoprostenol for patients with severe PAH. Scleroderma renal crisis necessitates the immediate use of angiotensin-converting enzyme inhibitors. Gastrointestinal manifestations are addressed with proton pump inhibitors for reflux, prokinetic agents for dysmotility and cyclical antibiotics in the setting of small intestinal bacterial overgrowth. Cutaneous fibrosis may be addressed with immunosuppressive therapies such as Methotrexate, Mycophenolate mofetil, Rituximab or Tocilizumab. Management of ILD involve immunosuppressive therapies such as Mycophenolate mofetil, Cyclophosphamide, Rituximab or Tocilizumab, as well as antifibrotic treatment with Nintedanib. Musculoskeletal manifestations are generally addressed through treatment with Methotrexate [6].<br>Even with the significant progress in the management of SSc over the past few decades, the disease continues to have substantial morbidity and mortality. Current management protocols have greatly improved survival in SSc-related ILD and PAH, but they are still limited regarding Raynaud phenomenon, digital ulcers, cardiac, gastrointestinal or renal involvement. Moreover, a segment of patients is not responsive to current therapies and experiences disease progression despite the appropriate therapy [7].<br>A survey on herbal therapies conducted with patients with SSc reported that nine of the 23 patients used of a variety of phytotherapy products (essential oils, herbal tea, vegetable oils or gemmotherapy). On a rating scale of 1-10, the perceived effectiveness of these treatments was an average of 7.2 [8].	Introduction, paragraphs 5-7
Objectives	4	In light of these findings, this research sought to evaluate the available data on the therapeutic potential of plant—derived agents in SSc, and to summarize the putative molecular mechanisms that might account for their bioactivity.	
**METHODS**			

Section and Topic	Item #	Checklist item	Location where item is reported
Eligibility criteria	5	Inclusion criteria for this review were established though a systemic search strategy combining various keywords including (“systemic sclerosis” OR “systemic scleroderma” OR “scleroderma” OR “sclerosis”) AND (“plants” OR “herb” OR “phytocompound” OR “phytochemicals”) NOT ("multiple sclerosis" OR "fungus" OR "lateral sclerosis" OR "tuberous sclerosis"). Additionally, a linguistic eligibility criterion was implemented to include only studies published in the English language.<br>Exclusion criteria encompassed duplicate publication, studies addressed a natural compound but not related to systemic sclerosis, research on systemic sclerosis unrelated to natural compound and articles referred to herbal remedies from traditional medicine that had not been scientifically evaluated for systemic sclerosis.	Matherials and Methods, paragrpahs 2-3
Information sources	6	The first step of our analysis consisted in a comprehensive search of the scientific literature, which was conducted in PubMed and ScienceDirect, encompassing publications from January 1975 through August 2025, with a final update in September 2025.	Matherials and Methods, paragrpah 1
Search strategy	7	To identify relevant articles, the search was performed combining various keywords including (“systemic sclerosis” OR “systemic scleroderma” OR “scleroderma” OR “sclerosis”) AND (“plants” OR “herb” OR “phytocompound” OR “phytochemicals”) NOT ("multiple sclerosis" OR "fungus" OR "lateral sclerosis" OR "tuberous sclerosis").	Matherials and Methods, paragrpah 2
Selection process	8	Two independent reviewers were involved in screening each record and report retrieved for inclusion in the review. Reviewers worked independently during the initial screening phase. Any discrepancies or disagreements regarding the eligibility of a study were resolved through discussion and consensus.	
Data collection process	9	Two independent reviewers were responsible for collecting data from each included report in the review. Reviewers worked independently during the data collection phase. Any discrepancies or disagreements were resolved through discussion and consensus. Data extraction was performed manually, and no automation tools were used in the process. This manual approach was chosen to ensure a detailed and accurate extraction of relevant information from each report.	
Data items	10a	In this systematic review, data were sought for the following outcomes:<br>**Primary Outcomes**:<br>• Phytocompounds<br>• Plant extracts<br>**Secondary Outcomes:**<br>• Molecular targets involved in systemic sclerosis pathogenesis<br>All results compatible with each outcome domain in each study were sought. The decision to include all relevant phytocompounds and plant extracts investigated in relation to systemic sclerosis, as well as all reported molecular targets related to these plant-based compounds, was made to ensure comprehensive assessment of their therapeutic potential and underlying mechanism.	Matherials and Methods, paragrpahs 2-5
	10b	In addition to the primary and secondary outcomes, data were sought for the following variables: Type of study: *in vitro, in vivo or human study*;	Tables1-6
Study risk of bias assessment	11	Risk of bias was assessed according to study design, using the Systematic Review Centre for Laboratory animal Experimentation (SYRCLE) Risk of Bias tool [11] for *in vivo* studies and the Risk Of Bias In Non-randomized Studies – of Interventions (ROBINS-I) tool for human studies [12].	Matherials and Methods, paragrpah 4
Effect measures	12	Not applicable - Narrative synthesis without meta-analysis. Results presented as qualitative descriptions of effects (↓, ↑ symbols with dose-dependent responses	

Section and Topic	Item #	Checklist item	Location where item is reported
Synthesis methods	13a	The main characteristics of the studies included in this systematic review are presented in Supplementary Table S1. An abbreviated version of the Standard Quality Assessment Criteria by Kmet et al. [10] was used to evaluate the methodological quality of all studies.	Matherials and Methods, paragrpah 4
	13b	Information from studies employing diverse methodologies was aligned through data harmonization. Variables measured in different units were standardized or converted to a common scale for consistency.	
	13c	Lastly, 32 studies (original or research articles) were selected for the review and were included in the synthesized tables (Tables 1-6).	Results, paragraph 1
	13d	Results were synthesized using a qualitative (narrative) approach, supported by structured tabular summaries. A quantitative meta-analysis was not performed due to the substantial heterogeneity among the included studies in terms of study design (in vitro, in vivo, and human studies), experimental models, patient populations, interventions, and outcome measures. Given this variability, statistical pooling was considered inappropriate. Therefore, findings were grouped and discussed according to study type and level of evidence, focusing on mechanistic pathways in preclinical studies and clinical outcomes in human studies.	
	13e	Potential sources of heterogeneity were explored qualitatively by considering differences in study design, methodological quality, and outcome measures during narrative interpretation.	
	13f	No formal sensitivity analyses were conducted, as a quantitative synthesis or meta-analysis was not performed. Methodological quality and risk of bias assessments were used to inform qualitative interpretation of the findings rather than to conduct robustness testing.	
Reporting bias assessment	14	Risk of bias due to missing results (reporting bias) was assessed qualitatively during study selection and risk of bias evaluation. No studies were excluded on the basis of selective reporting, as none were judged to have a high risk of reporting bias according to the applied assessment tools.	
Certainty assessment	15	No formal framework (such as GRADE) was used to assess certainty in the body of evidence. Given the predominantly preclinical nature of the included studies, the heterogeneity of study designs and outcomes, and the narrative synthesis approach, formal certainty grading was not considered applicable. Confidence in the evidence was instead addressed qualitatively by considering study design, methodological quality, and risk of bias during interpretation. shorten this	
**RESULTS**			
Study selection	16a	Figure 2	Results
	16b	Some exemples of excluded articles:<br>1. Bylka W, Znajdek-Awiżeń P, Studzińska-Sroka E, Brzezińska M. Centella asiatica in cosmetology. *Postepy Dermatol Alergol*. 2013;30(1):46-49. doi:10.5114/pdia.2013.33378 - **it is a review, does not include patients/murine models of systemic sclerosis**<br>2. Hong SS, Kim JH, Li H, Shim CK. Advanced formulation and pharmacological activity of hydrogel of the titrated extract of C. asiatica. Arch Pharm Res. 2005 Apr;28(4):502-8. doi: 10.1007/BF02977683. PMID: 15918527. - **it is a review, does not include patients/murine models of systemic sclerosis**<br>3. Salehi B, Rescigno A, Dettori T, Calina D, Docea AO, Singh L, Cebeci F, Özçelik B, Bhia M, Dowlati Beirami A, Sharifi-Rad J, Sharopov F, Cho WC, Martins N. Avocado-Soybean Unsaponifiables: A Panoply of Potentialities to Be Exploited. Biomolecules. 2020 Jan 13;10(1):130. doi: 10.3390/biom10010130. PMID: 31940989; PMCID: PMC7023362. - **it is a review, does not include patients/murine**<br>4. Gaby AR. Natural remedies for scleroderma. Altern Med Rev. 2006 Sep;11(3):188-95. PMID: 17217320. - **it is a review, does not include patients/ murine models of systemic sclerosis**<br>5. Xian D, Guo M, Xu J, Yang Y, Zhao Y, Zhong J. Current evidence to support the therapeutic potential of flavonoids in oxidative	

Section and Topic	Item #	Checklist item	Location where item is reported
		stress-related dermatoses. Redox Rep. 2021 Dec;26(1):134-146. doi: 10.1080/13510002.2021.1962094. PMID: 34355664; PMCID: PMC8354022. - **it is a review, does not include patients/ murine models of systemic sclerosis**<br>6. Bandopadhyay S, Mandal S, Ghorai M, Jha NK, Kumar M, Radha, Ghosh A, Proćków J, Pérez de la Lastra JM, Dey A. Therapeutic properties and pharmacological activities of asiaticoside and madecassoside: A review. J Cell Mol Med. 2023 Mar;27(5):593-608. doi: 10.1111/jcmm.17635. Epub 2023 Feb 8. PMID: 36756687; PMCID: PMC9983323. - **it is a review, does not include patients/ murine models of systemic sclerosis**	
Study characteristics	17	Supplementary table S1. The main characteristics of the studies included in this systematic review	
Risk of bias in studies	18	Supplementary Table S3 – Risk of bias assessed using the Systematic Review Centre for Laboratory animal Experimentation (SYRCLE) Risk of Bias tool<br>Supplementary Table S4 – Risk of bias assessed using the Risk Of Bias In Non-randomized Studies – of Interventions (ROBINS-I) tool	Supplementary materials
Results of individual studies	19	N/A	
Results of syntheses	20a	Contributing studies were heterogeneous in design, with generally low to moderate risk of bias as assessed using appropriate study-specific tools.	
	20b	N/A	
	20c	Qualitative assessment indicated that heterogeneity among study results was primarily attributable to differences in study design and experimental context, including in vitro, in vivo murine, and human studies. Additional sources of variability included differences in experimental models, interventions, dosing regimens, and outcome measures. Human studies further differed in patient populations, disease subsets, and clinical endpoints assessed. No consistent patterns of heterogeneity were attributable to methodological quality or risk of bias.	
	20d	N/A	
Reporting biases	21	Our search strategy was designed to be comprehensive, encompassing multiple databases<br>The quality of reporting in each included study was systematically evaluated using PRISMA method.	
Certainty of evidence	22	Formal assessments of certainty (e.g., outcome-specific confidence ratings) were not performed for individual outcomes. Given the heterogeneity of outcomes, study designs, and the predominance of preclinical evidence, certainty in the body of evidence was addressed qualitatively. Confidence was informed by study design, consistency of findings across experimental models, methodological quality, and risk of bias, with preclinical mechanistic evidence interpreted as hypothesis-generating and human data considered preliminary.	
**DISCUSSION**			
Discussion	23a	The evidence summarized in this review spans a broad spectrum of experimental models and clinical contexts. Most available data derive from preclinical studies, including *in vitro* experiments and murine models of systemic sclerosis, which primarily provide mechanistic insights into antifibrotic, anti-inflammatory and antioxidant pathways relevant to SSc. In contrast, human studies mainly report clinical outcome improvements, such as changes in the MRSS, frequency and severity of Raynaud phenomenon, hand mobility, or respiratory function parameters, with limited mechanistic exploration. A major limitation of the available literature is therefore the predominance of preclinical data, with relatively few clinical studies. Accordingly, findings from preclinical and human studies were discussed separately to reflect differences in evidentiary depth; preclinical observations establish biological plausibility and mechanistic rationale, while the available human data represent an initial step toward clinical translation. Overall, the current evidence should be interpreted as hypothesis-generating rather than practice-changing, underscoring the need for well-designed clinical studies.	Discussion, paragraph 18

PRISMA 2020 Checklist

Section and Topic	Item #	Checklist item	Location where item is reported
	23b	The review was limited to studies published in English, introducing the potential for language bias.	
	23c	The search was limited to specific databases, potentially missing relevant studies in other sources	
	23d	Other future research direction might be to identify potential synergisms between these phytochemicals and standard conventional therapy and to analyze their putative utility as complementary therapy in SSc. Their extensive structural optimization may be also potentially useful in designing new drugs with clinical therapeutic efficacy in SSc. The safety and potential long-term effects of these phytochemicals or plant extracts remain to be established, as they have not been investigated in most cases.	Further direction of research – paragraphs 6-8
**OTHER INFORMATION**			
Registration and protocol	24a	The systematic review was not registered in any systematic review registry.	
	24b	A review protocol was not prepared for this study.	
	24c	N/A	
Support	25	This research received no external funding.	
Competing interests	26	The authors declare no conflict of interest	
Availability of data, code and other materials	27	Please contact the corresponding author for any inquiries regarding data access.	

From: Page MJ, McKenzie JE, Bossuyt PM, Boutron I, Hoffmann TC, Mulrow CD, et al. The PRISMA 2020 statement: an updated guideline for reporting systematic reviews. BMJ 2021;372:n71. doi: 10.1136/bmj.n71. This work is licensed under CC BY 4.0. To view a copy of this license, visit https://creativecommons.org/licenses/by/4.0/
