# Supplementary material for: Plant-Derived Agents and Systemic Sclerosis: A Systematic Review of Therapeutic Potential and Molecular Mechanisms
Source: Curr Issues Mol Biol. 2026 Jan 18;48(1):97. doi: 10.3390/cimb48010097 (PMC12840015; doi:10.3390/cimb48010097)
Supplement: Supplementary file 1 [file cimb-48-00097-s001.zip › Supplementary materials_C2.pdf]

Search strategy using Bollean operators

The search strategies were based primarily on keyword searches using Boolean operators. The following reflects the practical search approach conducted across both databases.

PubMed Search Strategy (as conducted)

“systemic sclerosis” AND “plants” NOT ("multiple sclerosis" OR "fungus" OR "lateral sclerosis" OR "tuberous sclerosis")

“systemic scleroderma” AND “plants” NOT ("multiple sclerosis" OR "fungus" OR "lateral sclerosis" OR "tuberous sclerosis")

“scleroderma” AND “plants” NOT ("multiple sclerosis" OR "fungus" OR "lateral sclerosis" OR "tuberous sclerosis")

“sclerosis” AND “plants” NOT ("multiple sclerosis" OR "fungus" OR "lateral sclerosis" OR "tuberous sclerosis")

“systemic sclerosis” AND “herb” NOT ("multiple sclerosis" OR "fungus" OR "lateral sclerosis" OR "tuberous sclerosis")

“systemic scleroderma” AND “herb” NOT ("multiple sclerosis" OR "fungus" OR "lateral sclerosis" OR "tuberous sclerosis")

“scleroderma” AND “herb” NOT ("multiple sclerosis" OR "fungus" OR "lateral sclerosis" OR "tuberous sclerosis")

“sclerosis” AND “herb” NOT ("multiple sclerosis" OR "fungus" OR "lateral sclerosis" OR "tuberous sclerosis")

“systemic sclerosis” AND “phytocompound” NOT ("multiple sclerosis" OR "fungus" OR "lateral sclerosis" OR "tuberous sclerosis")

“systemic scleroderma” AND “phytocompound” NOT ("multiple sclerosis" OR "fungus" OR "lateral sclerosis" OR "tuberous sclerosis")

“scleroderma” AND “phytocompound” NOT ("multiple sclerosis" OR "fungus" OR "lateral sclerosis" OR "tuberous sclerosis")

“sclerosis” AND “phytocompound” NOT ("multiple sclerosis" OR "fungus" OR "lateral sclerosis" OR "tuberous sclerosis")

“systemic sclerosis” AND “phytochemicals” NOT ("multiple sclerosis" OR "fungus" OR "lateral sclerosis" OR "tuberous sclerosis")

“systemic scleroderma” AND “phytochemicals” NOT ("multiple sclerosis" OR "fungus" OR "lateral sclerosis" OR "tuberous sclerosis")

“scleroderma” AND “phytochemicals” NOT ("multiple sclerosis" OR "fungus" OR "lateral sclerosis" OR "tuberous sclerosis")

“sclerosis” AND “phytochemicals” NOT ("multiple sclerosis" OR "fungus" OR "lateral sclerosis" OR "tuberous sclerosis")

Filters applied: English language

ScienceDirect Search Strategy (as conducted)

“systemic sclerosis” AND “plants” NOT ("multiple sclerosis" OR "fungus" OR "lateral sclerosis" OR "tuberous sclerosis")

“systemic scleroderma” AND “plants” NOT ("multiple sclerosis" OR "fungus" OR "lateral sclerosis" OR "tuberous sclerosis")

“scleroderma” AND “plants” NOT ("multiple sclerosis" OR "fungus" OR "lateral sclerosis" OR "tuberous sclerosis")

“sclerosis” AND “plants” NOT ("multiple sclerosis" OR "fungus" OR "lateral sclerosis" OR "tuberous sclerosis")

“systemic sclerosis” AND “herb” NOT ("multiple sclerosis" OR "fungus" OR "lateral sclerosis" OR "tuberous sclerosis")

“systemic scleroderma” AND “herb” NOT ("multiple sclerosis" OR "fungus" OR "lateral sclerosis" OR "tuberous sclerosis")

“scleroderma” AND “herb” NOT ("multiple sclerosis" OR "fungus" OR "lateral sclerosis" OR "tuberous sclerosis")

“sclerosis” AND “herb” NOT ("multiple sclerosis" OR "fungus" OR "lateral sclerosis" OR "tuberous sclerosis")

“systemic sclerosis” AND “phytocompound” NOT ("multiple sclerosis" OR "fungus" OR "lateral sclerosis" OR "tuberous sclerosis")

“systemic scleroderma” AND “phytocompound” NOT ("multiple sclerosis" OR "fungus" OR "lateral sclerosis" OR "tuberous sclerosis")

“scleroderma” AND “phytocompound” NOT ("multiple sclerosis" OR "fungus" OR "lateral sclerosis" OR "tuberous sclerosis")

“sclerosis” AND “phytocompound” NOT ("multiple sclerosis" OR "fungus" OR "lateral sclerosis" OR "tuberous sclerosis")

“systemic sclerosis” AND “phytochemicals” NOT ("multiple sclerosis" OR "fungus" OR "lateral sclerosis" OR "tuberous sclerosis")

“systemic scleroderma” AND “phytochemicals” NOT ("multiple sclerosis" OR "fungus" OR "lateral sclerosis" OR "tuberous sclerosis")

“scleroderma” AND “phytochemicals” NOT ("multiple sclerosis" OR "fungus" OR "lateral sclerosis" OR "tuberous sclerosis")

“sclerosis” AND “phytochemicals” NOT ("multiple sclerosis" OR "fungus" OR "lateral sclerosis" OR "tuberous sclerosis")

Filters applied: English language, Research articles

These search strings reflect the actual process undertaken in this review and are provided for transparency and reproducibility.

Supplementary table S1. The main characteristics of the studies included in this systematic review

**Article**	**First author**	**Year of publication**	**No of references**	**Type of study**	**Control lot**
Inhibition of collagen type I synthesis by skin fibroblasts of graft versus host disease and scleroderma patients: Effect of halofuginone	Halevy Orna	1996	11	*In vitro* – fribroblasts from 3 SSc patients and 3 HS	Yes
Antifibrotic effects of crocetin in scleroderma fibroblasts and in bleomycin-induced sclerotic mice.	Yinghua Song	2013	12	*In vitro* – fribroblasts from 3 SSc patients and 3 HS	Yes
				In vivo – six-week-old female mice	Yes
Tanshinone IIA attenuates interleukin-17A-induced systemic sclerosis patient-derived dermal vascular smooth muscle cell activation via inhibition of the extracellular signal-regulated kinase signaling pathway	Mengguo Liu	2015	13	*In vitro* – DVSMCs from 10 SSc patients	No
Tanshinone IIA ameliorates the bleomycin-induced endothelial-to-mesenchymal transition via the Akt/mTOR/p70S6K pathway in a murine model of systemic sclerosis	Ying Jiang	2019	14	*In vitro – HUVECs*	Yes
				In vivo – 48 six-week-old female mice	Yes
Resveratrol ameliorates systemic sclerosis via suppression of fibrosis and inflammation through activation of sirt1/mtor signaling	Qicen Yao	2020	15	*In vitro* – fribroblasts from 10 SSc patients and 10 HS	Yes

Inhibition of collagen hydroxylation by lithospermic acid magnesium salt, a novel compound isolated from Salviae miltiorrhizae Radix	Tomohiro Shigematsu	1994	16	*In vitro* – fibroblasts from HS	Yes
				In vivo – 6 ten-week-old male hairless mice	Yes
The natural organosulfur compound dipropyltetrasulfide prevents HOCl-induced systemic sclerosis in the mouse	Wioleta Marut	2013	17	*In vitro* – fibroblasts from mice which recived or not injections with HOCl generating	Yes
				In vivo – Six-week-old female mice	Yes
Geniposide inhibited endothelial-mesenchymal transition via the mTOR signaling pathway in a bleomycin-induced scleroderma mouse model	Qing Qi	2017	18	*In vitro – HUVECs*	Yes
				In vivo – female mice	Yes
Abscisic acid ameliorates the systemic sclerosis fibroblast phenotype in vitro	Santina Bruzzone	2012	21	*In vitro* – fribroblasts from 11 SSc patients and 9 HS	Yes
A novel inhibitor of Smad-dependent transcriptional activation suppresses tissue fibrosis in mouse models of systemic sclerosis	Minoru Hasegawa	2009	23	*In vitro – fibroblasts from HS*	Yes
				In vivo – Four-week-old female tight skin mice	Yes

Inhibitory effect of kaempferol on skin fibrosis in systemic sclerosis by the suppression of oxidative stress	Akiko Sekiguchi	2019	24	*In vitro* – fribroblasts from 3 SSc patients and 3 HS	Yes
				In vivo – Eight-week-old mice	Yes
Celastrol is a novel selective agonist of cannabinoid receptor 2 with anti-inflammatory and anti-fibrotic activity in a mouse model of systemic sclerosis	Xingwu Jiang	2020	25	*In vitro* – Raw 264.7 cells and bone marrow-derived macrophages	Yes
				In vivo – Six-eight-old female mice	Yes
Modulation of collagen type I, fibronectin and dermal fibroblast function and activity, in systemic sclerosis by the antioxidant epigallocatechin-3-gallate	Audrey Dooley	2010	26	*In vitro* – fribroblasts from 8 SSc patients and 8 HS	Yes
Dihydromyricetin, the active component of rattan tea alleviates symptoms of systemic sclerosis and atopic dermatitis through modulation of RORγt and IL17A production in T cells	Debanjan Sarkar	2025	27	*In vitro – helper T cells*	Yes
				In vivo – Eight-weeks-old mice	Yes
Curcumin suppresses TGF-β signaling by inhibition of TGIF degradation in scleroderma fibroblasts	Kemin Song	2011	29	*In vitro – firbroblasts from 5 SSc patients*	Yes
Inhibitory effects of ursolic acid from Bushen Yijing Formula on TGF-β1-induced human umbilical vein	Ke Zhu	2019	30	*In vitro – HUVECs*	Yes

endothelial cell fibrosis via AKT/mTOR signaling and Snail gene					
Verbascoside and isoverbascoside ameliorate transforming growth factor β1-induced collagen expression by lung fibroblasts through Smad/non-Smad signaling pathways	Chung-Yu Chen	2022	31	*In vitro* – murine normal lung fibroblasts cell line MLg 2908 and human primary pulmonary cells	Yes
Activin, a grape seed-derived proanthocyanidin extract, reduces plasma levels of oxidative stress and adhesion molecules (ICAM-1, VCAM-1 and E-selectin) in systemic sclerosis	Reni Kalin	2002	32	*Human – SSc patients and HS*	Yes
Pineapple Proteases in the Treatment of Scleroderma: A Case Report	Pierce H E Jr.	1964	33	*Human – SSc patient*	No
Nimbolide ameliorates fibrosis and inflammation in experimental murine model of bleomycin-induced scleroderma	Snehalatha Diddi	2019	34	*In vivo* – 7-eight-weeks-old male mice	Yes
Withaferin A attenuates bleomycin-induced scleroderma by targeting FoxO3a and NF-κβ signaling: Connecting fibrosis and inflammation	Swarna Bale	2018	35	*In vivo – eight-*nine-week-old male mice	Yes
Astragalus polysaccharide suppresses excessive collagen accumulation in a murine model of bleomycin-induced scleroderma	Zhen-Feng Hao	2015	36	*In vivo* – Six-weeks-old mice	Yes
Madecassoside Ameliorates Bleomycin-Induced Pulmonary Fibrosis in Mice by Downregulating Collagen Deposition	Guo-Xun Lu	2014	37	*In vivo* – *f*emale mice	Yes
Asiaticoside might attenuate bleomycin-induced pulmonary fibrosis by activating cAMP and Rap1 signalling pathway assisted by A2AR	Jing Luo	2020	38	*In vivo* – 45 wild-type mice and 45 Adenosine 2A	Yes

				receptor gene knockout mice	
From a Designer Drug to the Discovery of Selective Cannabinoid Type 2 Receptor Agonists with Favorable Pharmacokinetic Profiles for the Treatment of Systemic Sclerosis	Bei-Er Jiang	2021	39	*In vivo – mice*	
Capparis spinosa protects against oxidative stress in systemic sclerosis dermal fibroblasts	Yue-Lan Cao	2010	41	*In vitro* – fibroblasts from 3 SSc patients and 3 HS	Yes
Capparis spinosa inhibits proliferation and fibrosis of myofibroblasts in systemic sclerosis through modulation of MAPK signaling	Xin Xin Qi	2025	42	*In vitro* – lung fibroblasts isolated from mice	Yes
				In vivo – Eight-weeks-old mice	Yes
A Double-blind, Randomized Controlled Trial of Ciplukan (Physalis angulata Linn) Extract on Skin Fibrosis, Inflammatory, Immunology, and Fibrosis Biomarkers in Scleroderma Patients	Sumartini Dewi	2019	43	*Human* – 59 SSc patients	Yes
The Chinese herb Tripterygium wilfordii Hook F for the treatment of systemic sclerosis-associated interstitial lung disease: data from a Chinese EUSTAR Center	Luwei Yang	2020	44	*Human* – 76 SSc patients with ILD	Yes
Results of a Pilot Randomized Placebo-Controlled Trial in Primary and Secondary Raynaud's Phenomenon with St. John's Wort: Detecting Changes in Angiogenic Cytokines When RP Improves	Deanne Malenfant	2011	46	*Human* – patients with primary Raynaud`s phenomenon and secondary	Yes

				Raynaud`s phenomenon associated with SSc or other connective tissue diseases	
Evening primrose oil (Efamol) in the treatment of Raynaud's phenomenon: a double blind study	Belch J J	1985	47	*Human* – 21 patients with Raynaud`s phenomenon with or without SS	
Clinical aspects of the use of gamma linolenic acid in systemic sclerosis	Stainforth J M	1996	48	*Human* – 25 SSc patients	

SSc – systemic sclerosis; HS – healthy subjects; HUVECs – human umbilical vein endothelial cells; DVSMCs – Dermal vascular smooth muscle cells

Supplementary table S2. Quality assessment of all studies evaluated using an abbreviated version of the Standard Quality Assessment Criteria for Evaluating Primary Research Papers from a Variety of Fields developed by Kmet et al.

	Q1	Q2	Q3	Q4	Q5	Q6	Q7	Q8	Q9	Q10	Q11	Q12
Halevy Orna	Y	Y	Y	Y	N/A	N/A	N/A	Y	P	Y	Y	Y
Yinghua Song	Y	Y	Y	Y	N/A	N/A	N/A	Y	P	Y	Y	Y
Mengguo Liu	Y	Y	Y	Y	N/A	N/A	N/A	Y	P	Y	Y	Y
Ying Jiang	Y	Y	Y	Y	P	N/A	N/A	Y	Y	Y	Y	Y
Qicen Yao	Y	Y	Y	Y	N/A	N/A	N/A	Y	P	Y	Y	Y
Tomohiro Shigematsu	Y	Y	Y	Y	N/A	N/A	N/A	Y	P	Y	Y	Y
Wioleta Marut	Y	Y	Y	Y	N/A	P	N/A	Y	P	Y	Y	Y
Qing Qi	Y	Y	Y	Y	P	P	N/A	Y	P	Y	Y	Y
Santina Bruzzone	Y	Y	Y	Y	N/A	N/A	N/A	Y	P	Y	Y	Y
Minoru Hasegawa	Y	Y	Y	Y	P	N/A	N/A	Y	P	Y	Y	Y
Akiko Sekiguchi	Y	Y	Y	Y	N/A	N/A	N/A	Y	P	Y	Y	Y
Xingwu Jiang	Y	Y	Y	Y	P	N/A	N/A	Y	P	Y	Y	Y
Audrey Dooley	Y	Y	Y	Y	N/A	N/A	N/A	Y	P	Y	Y	Y

Debanjan Sarkar	Y	Y	Y	Y	N/A	N/A	N/A	Y	P	Y	Y	Y
Kemin Song	Y	Y	Y	Y	N/A	N/A	N/A	Y	P	Y	Y	Y
Ke Zhu	Y	Y	Y	Y	N/A	N/A	N/A	Y	P	Y	Y	Y
Chung-Yu Chen	Y	Y	Y	Y	N/A	N/A	N/A	Y	P	Y	Y	Y
Reni Kalin	Y	Y	Y	Y	Y	Y	Y	Y	Y	Y	Y	Y
Pierce H E Jr.	Y	N/A	Y	Y	N/A	N/A	N/A	Y	N/A	Y	N	Y
Snehalatha Diddi	Y	Y	Y	Y	P	N/A	N/A	Y	Y	Y	Y	Y
Swarna Bale	Y	Y	Y	Y	P	N/A	N/A	Y	Y	Y	Y	Y
Zhen-Feng Hao	Y	Y	Y	Y	P	P	N/A	Y	Y	Y	Y	Y
Guo-Xun Lu	Y	Y	Y	Y	P	P	N/A	Y	P	Y	Y	Y
Jing Luo	Y	Y	Y	Y	P	N/A	N/A	Y	Y	Y	Y	Y
Bei-Er Jiang	Y	Y	Y	Y	N/A	N/A	N/A	Y	P	Y	Y	Y
Yue-Lan Cao	Y	Y	Y	Y	N/A	N/A	N/A	Y	P	Y	Y	Y
Xin Xin Qi	Y	Y	Y	Y	N/A	N/A	N/A	Y	P	Y	Y	Y
Sumartini Dewi	Y	Y	Y	Y	Y	P	P	Y	Y	Y	P	Y
Luwei Yang	Y	Y	Y	Y	N/A	N/A	N/A	Y	Y	Y	P	Y
Deanne Malenfant	Y	Y	Y	Y	Y	Y	Y	Y	P	Y	P	Y
Belch J J	Y	Y	Y	Y	P	P	P	Y	P	Y	P	Y
Stainforth J M	Y	Y	Y	Y	P	P	P	Y	P	Y	P	Y

Q1 Question or objective sufficiently described?; Q2 Design appropriate to answer study questions?; Q3 Were the source of information appropriate and well described?; Q4 Were the units of participation sufficiently described?; Q5 If random allocation to treatment group was done, is it well described?; Q6 If interventional and blinding of investigators to intervention present, is it well described?; Q7 If interventional and blinding of subjects to intervention present, is it well described?; Q8 Were the means of assessment reported?; Q9 Sample size was appropriate?; Q10 Were analytic methods well described and appropriate? Q11 Controlled for confounding? Q12 Are the conclusions supported by the results?
Y – Yes, P - Partial, N – No, N/A – Not Applicable

Supplementary Table S3 – Risk of bias assessed using the Systematic Review Centre for Laboratory animal Experimentation (SYRCLE) Risk of Bias tool

	Q1	Q2A	Q2B	Q2C	Q3	Q4A	Q4B	Q5	Q6	Q7A	Q7B	Q8A	Q8B	Q8C	Q8D	Q9A	Q9B	Q10A	Q10B	Q10C	Q10D	Q10E
Yinghua Song	N	Y	N/A	Y	U	U	Y	N	Y	U	N	Y	N/A	N/A	N/A	Y	N/A	Y	U	Y	Y	N/A
Ying Jiang	Y	Y	N/A	Y	Y	U	Y	N	U	U	N	Y	N/A	N/A	N/A	Y	N/A	Y	U	Y	Y	N/A

Tomohiro Shigematsu	N	Y	N/A	N/A	U	U	Y	N	U	U	N	Y	N/A	N/A	N/A	Y	N/A	Y	Y	Y	Y	N/A
Wioleta Marut	N	Y	N/A	Y	U	U	Y	N	U	U	N	Y	N/A	N/A	N/A	Y	N/A	Y	U	Y	Y	N/A
Qing Qi	Y	Y	N/A	Y	Y	U	Y	N	U	U	N	Y	N/A	N/A	N/A	Y	N/A	Y	U	Y	Y	N/A
Minoru Hasegawa	Y	Y	N/A	Y	Y	U	Y	N	U	U	N	Y	N/A	N/A	N/A	Y	N/A	Y	Y	Y	Y	N/A
Akiko Sekiguchi	N	Y	N/A	Y	U	U	Y	N	U	U	N	Y	N/A	N/A	N/A	Y	N/A	Y	Y	Y	Y	N/A
Xingwu Jiang	Y	Y	N/A	Y	Y	U	Y	N	U	U	N	Y	N/A	N/A	N/A	Y	N/A	Y	U	Y	Y	N/A
Debanjan Sarkar	Y	Y	N/A	Y	Y	U	Y	N	U	U	N	Y	N/A	N/A	N/A	Y	N/A	Y	U	Y	Y	N/A
Snehalatha Diddi	Y	Y	N/A	Y	Y	U	Y	N	U	U	N	Y	N/A	N/A	N/A	Y	N/A	Y	U	Y	Y	N/A
Swarna Bale	Y	Y	N/A	Y	Y	U	Y	N	U	U	N	Y	N/A	N/A	N/A	Y	N/A	Y	U	Y	Y	N/A
Zhen-Feng Hao	Y	Y	N/A	Y	Y	U	Y	N	U	U	N	Y	N/A	N/A	N/A	Y	N/A	Y	U	Y	Y	N/A
Guo-Xun Lu	Y	Y	N/A	Y	Y	U	Y	N	U	U	N	Y	N/A	N/A	N/A	Y	N/A	Y	U	Y	Y	N/A
Jing Luo	Y	Y	N/A	Y	Y	U	Y	N	U	U	N	Y	N/A	N/A	N/A	Y	N/A	Y	U	Y	Y	N/A
Bei-Er Jiang	N	Y	N/A	Y	U	U	Y	N	U	U	N	Y	N/A	N/A	N/A	Y	N/A	Y	U	Y	Y	N/A
Xin Xin Qi	N	Y	N/A	Y	U	U	Y	N	U	U	N	Y	N/A	N/A	N/A	Y	N/A	Y	U	Y	Y	N/A

Q1 - Was the allocation sequence adequately generated and applied?; Q2A - Was the distribution of relevant baseline characteristics balanced for the intervention and control groups?; Q2B - If relevant, did the investigators adequately adjust for unequal distribution of some relevant baseline characteristics in the analysis?; Q2C - Was the timing of disease induction adequate?; Q3 - Was the allocation to the different groups adequately concealed during?; Q4A - Did the authors randomly place the cages or animals within the animal room/facility?; Q4B - Is it unlikely that the outcome or the outcome measurement was influenced by not randomly housing the animals?; Q5 - Were the caregivers and/or investigators blinded from knowledge which intervention each animal received during the experiment?; Q6 - Were animals selected at random for outcome assessment?; Q7A - Was blinding of the outcome assessor ensured, and was it unlikely that blinding could have been broken?; Q7B - Was the outcome assessor not blinded, but do review authors judge that the outcome is not likely to be influenced by lack of blinding?; Q8A - Were all animals included in the analysis?; Q8B - Were the reasons for missing outcome data unlikely to be related to true outcome? (e.g., technical failure); Q8C - Are missing outcome data balanced in numbers across intervention groups, with similar reasons for missing data across groups?; Q8D - Are missing outcome data imputed using appropriate methods?; Q9A - Was the study protocol available and were all of the study's pre-specified primary and secondary outcomes reported in the current manuscript?; Q9B - Was the study protocol not available, but was it clear that the published report included all expected outcomes (i.e. comparing methods and results section)?; Q10A - Was the study free of contamination (pooling drugs)?; Q 10B - Was the study free of inappropriate influence of funders?; Q10C - Was the study free of unit of analysis errors?; Q 10D - Were design-specific risks of bias absent?; Q10E - Were new animals added to the control and experimental groups to replace drop-outs from the original population?; Y – yes; N – no; U – unclear; N/A – not applicable

Supplementary Table S4 – Risk of bias assessed using the Risk Of Bias In Non-randomized Studies – of Interventions (ROBINS-I) tool

	Domain 1	Domain 2	Domain 3	Domain 4	Domain 5	Domain 6

Reni Kalin	+	+	+	+	+	+
Sumartini Dewi	-	+	+	+	+	+
Luwei Yang	-	+	+	+	+	+
Deanne Malenfant	-	+	+	+	+	+
Belch J J	-	+	+	+	+	+
Stainforth J M	-	+	+	+	+	+

Domain 1 – Risk of bias due to confounding; Domain 2 – Risk of bias in classification of interventions; Domain 3 – Risk of bias in selection of participants into the study (or into the analysis); Domain 4 – Risk of bias due to missing data; Domain 5 – Risk of bias arising from measurment of the outcome; Domain 6 – Risk of bias in selection of the reported result; ,,+'' – low risk of bias; ,,-'' – moderate risk of bias; ,,x'' – serious risk of bias

Supplementary Table S6 – Putative molecular targets of the selected phytochemicals - identified with SwissTargetPrediction tool

**Molecular Targets**	**Common name**
11-beta-hydroxysteroid dehydrogenase 1	HSD11B1
6-phosphogluconate dehydrogenase	PGD
Acetylcholinesterase	ACHE
Acyl coenzyme A:cholesterol acyltransferase	CES1
Aldo-keto reductase family 1 member B10	AKR1B10
Aldose reductase	AKR1B1
Alpha-(1,3)-fucosyltransferase 7	FUT7
Apoptosis regulator Bcl-2	BCL2
Arachidonate 12-lipoxygenase	ALOX12
Arachidonate 15-lipoxygenase	ALOX15
Arachidonate 5-lipoxygenase	ALOX5
Aryl hydrocarbon receptor	AHR
ATP-binding cassette sub-family G member 2	ABCG2
Beta amyloid A4 protein	APP
Beta-secretase 1	BACE1
Carbonic anhydrase I	CA1
Carbonic anhydrase II	CA2

Carbonic anhydrase III	CA3
Carbonic anhydrase IV	CA4
Carbonic anhydrase IX	CA9
Carbonic anhydrase VA	CA5A
Carbonic anhydrase VB	CA5B
Carbonic anhydrase VI	CA6
Carbonic anhydrase VII	CA7
Carbonic anhydrase XII	CA12
Carbonic anhydrase XIII	CA13
Carbonic anhydrase XIV	CA14
Carboxylesterase 2	CES2
CMP-N-acetylneuraminate-beta-1,4-galactoside alpha-2,3-sialyltransferase	ST3GAL3
Cyclin-dependent kinase 1/cyclin B	CCNB3 CDK1 CCNB1 CCNB2
Cyclin-dependent kinase 5/CDK5 activator 1	CDK5R1 CDK5
Cyclooxygenase-1	PTGS1
Cyclooxygenase-2	PTGS2
Cytochrome P450 1A2	CYP1A2
Cytochrome P450 1B1	CYP1B1
Cytochrome P450 2C19	CYP2C19
Cytochrome P450 2C9	CYP2C9
Cytochrome P450 3A4	CYP3A4
Death-associated protein kinase 1	DAPK1
DNA (cytosine-5)-methyltransferase 1	DNMT1
DNA polymerase beta	POLB
DNA topoisomerase II alpha	TOP2A
DNA-3-methyladenine glycosylase	MPG
Dual specificity phosphatase Cdc25B	CDC25B
Dual-specificity tyrosine-phosphorylation regulated kinase 1A	DYRK1A
Estradiol 17-beta-dehydrogenase 1	HSD17B1
Estradiol 17-beta-dehydrogenase 2	HSD17B2
Estrogen receptor alpha	ESR1

Estrogen receptor beta	ESR2
Estrogen-related receptor alpha	ESRRA
Fucosyltransferase 4	FUT4
Glycogen synthase kinase-3 beta	GSK3B
Glyoxalase I	GLO1
G-protein coupled receptor 35	GPR35
Heat shock factor protein 1	HSF1
Hepatocyte growth factor receptor	MET
HERG	KCNH2
Histone acetyltransferase p300	EP300
Low molecular weight phosphotyrosine protein phosphatase	ACP1
MAP kinase p38 alpha	MAPK14
Matrix metalloproteinase 2	MMP12
Matrix metalloproteinase 9	MMP13
Matrix metalloproteinase 12	MMP14
Matrix metalloproteinase 13	MMP2
Matrix metalloproteinase 14	MMP9
Microtubule-associated protein tau	MAPT
Monoamine oxidase A	MAOA
Multidrug resistance-associated protein 1	ABCC1
NADPH oxidase 4	NOX4
Norepinephrine transporter	SLC6A2
Nuclear receptor ROR-gamma	RORC
P-glycoprotein 1	ABCB1
Phosphodiesterase 4D	PDE4D
Phospholipase A2 group 1B	PLA2G1B
PI3-kinase p110-alpha subunit	PIK3CA
PI3-kinase p110-beta subunit	PIK3CB
Prostaglandin E synthase	PTGES
Protein kinase C alpha	PRKCA
Protein-tyrosine phosphatase 1B	PTPN1

Protein-tyrosine phosphatase 1C	PTPN6
Protein-tyrosine phosphatase 2C	PTPN11
Quinone reductase 2	NQO2
Receptor-type tyrosine-protein phosphatase F (LAR)	PTPRF
Receptor-type tyrosine-protein phosphatase S	PTPRS
Signal transducer and activator of transcription 1-alpha/beta	STAT1
Solute carrier family 22 member 12	SLC22A12
T-cell protein-tyrosine phosphatase	PTPN2
Telomerase reverse transcriptase	TERT
Toll-like receptor (TLR7/TLR9)	TLR9
Tyrosinase	TYR
Tyrosine-protein kinase receptor FLT3	FLT3
Tyrosine-protein kinase SYK	SYK
Xanthine dehydrogenase	XDH
